# Islet amyloid polypeptide cross-seeds tau and drives the neurofibrillary pathology in Alzheimer’s disease

**DOI:** 10.1186/s13024-022-00518-y

**Published:** 2022-01-29

**Authors:** Guoxin Zhang, Lanxia Meng, Zhihao Wang, Qinyu Peng, Guiqin Chen, Jing Xiong, Zhentao Zhang

**Affiliations:** 1 Department of Neurology, Renmin Hospital of Wuhan University, Wuhan, 430060 China; 2 Department of Pathology and Laboratory Medicine, Emory University School of Medicine, Atlanta, GA 30322 USA

**Keywords:** Tauopathies, Type 2 diabetes, Islet amyloid polypeptide, Cross-seeding, Tau

## Abstract

**Background:**

The pathologic accumulation and aggregation of tau is a hallmark of tauopathies including Alzheimer’s disease (AD). However, the molecular mechanisms mediating tau aggregation in AD remain elusive. The incidence of AD is increased in patients with type 2 diabetes (T2DM), which is characterized by the amyloid deposition of islet amyloid polypeptide (IAPP) in the pancreas. However, the molecular mechanisms bridging AD and T2DM remain unknown.

**Methods:**

We first examined the presence of IAPP in the neurofibrillary tangles of AD patients. Then we tested the effect of IAPP on tau aggregation. The biochemical and biological characteristics of the IAPP-tau fibrils were tested in vitro. The seeding activity and neurotoxicity of the IAPP-tau fibrils were confirmed in cultured neurons. Lastly, the effect of IAPP on tau pathology and cognitive impairments was determined by injecting the IAPP-tau fibrils and IAPP fibrils into the hippocampus of tau P301S mice.

**Results:**

We found that IAPP interacts with tau and accelerates the formation of a more toxic strain, which shows distinct morphology with enhanced seeding activity and neurotoxicity in vitro. Intrahippocampal injection of the IAPP-tau strain into the tau P301S transgenic mice substantially promoted the spreading of tau pathology and induced more severe synapse loss and cognitive deficits, when compared with tau fibrils. Furthermore, intracerebral injection of synthetic IAPP fibrils initiated tauopathy in the brain of tau P301S transgenic mice.

**Conclusions:**

These observations indicate that IAPP acts as a crucial mediator of tau pathology in AD, and provide a mechanistic explanation for the higher risk of AD in individuals with T2DM.

**Supplementary Information:**

The online version contains supplementary material available at 10.1186/s13024-022-00518-y.

## Background

Tauopathies are a diverse group of neurodegenerative diseases defined by the aberrant aggregation and deposition of the microtubule-associated protein tau in the central nervous system. Alzheimer’s disease (AD) is the most common tauopathy and the most prevalent neurodegenerative disease. Tau is a microtubule-associated protein that is abundantly expressed in neurons. The physiological function of tau is to regulate microtubule polymerization and to stabilize microtubules. Under pathological conditions, tau aggregates into β-sheet structures and causes neurodegeneration and cognitive impairments. The prion hypothesis posits that the pathological tau aggregates act as templates to induce the aggregation of soluble tau, mediating the propagation of tau pathology throughout the brain [1]. Tau aggregates isolated from patients show distinct fibrillary structures and pathologic activities, which may account for the pathological and clinical heterogeneity in different tauopathies [2, 3]. However, the molecular mechanisms mediating the formation of different tau strains remain unclear.

Epidemiological evidence demonstrated a significant association between AD and Type 2 diabetes (T2DM) [4]. A community-based controlled study indicated that nearly 80% of AD patients exhibit glucose tolerance impairment or have been diagnosed with diabetes [5]. Vice versa, individuals with T2DM and insulin resistance also presented higher risks for developing mild cognitive impairment (MCI) and AD [6–9]. Pathologically, T2DM is characterized by the aberrant aggregation and deposition of the islet amyloid polypeptide (IAPP), which leads to the progressive degeneration of pancreatic islet β-cells [10, 11]. Mature IAPP is a peptide consisting of 37 amino acids, which is derived by proteolytic cleavage of the islet amyloid precursor protein. It is produced by pancreatic β-cells and co-released with insulin in response to high glucose levels [12].

Both AD and T2DM are considered as protein deposition diseases [13], of which the misfolded proteins organize in a β-sheet structure, leading to the transformation of soluble monomer to insoluble species, and accumulate in different tissues. An overlap of pathological characteristics in these diseases was frequently revealed, with one protein inducing the misfolding and aggregation of another [14, 15]. Recent findings from clinical and animal studies have implicated the involvement of IAPP in the deposition of pancreatic amyloid and in mediating cognitive dysfunction in AD [16, 17]. Besides, increased IAPP is detected in the cerebrospinal fluid (CSF) of AD patients with or without apparent T2DM [18], as well as in the temporal lobe gray matter in patients with T2DM [19, 20], indicating that circulating IAPP can cross the blood-brain barrier (BBB) and deposit in the brain. Meanwhile, amyloid-β (Aβ) and tau deposits have also been found in the pancreas of T2DM patients [21], indicating the potential pathological association of the two diseases. Furthermore, a positive correlation between retinal IAPP and hippocampal IAPP levels has been reported [22]. Interestingly, cross-amyloid interaction of Aβ and IAPP was detected at lipid membranes [23]. IAPP promotes the aggregation of Aβ in vitro in a seeding-like manner and forms fibrils that are composed of both peptides. However, it remains unknown whether IAPP is involved in tau pathology in AD.

In this report, we show that IAPP colocalizes with pathological tau in AD brain. IAPP binds tau and promotes its aggregation into a more toxic strain that shows increased seeding activity and neurotoxicity in vitro. Intrahippocampal injection of the IAPP-modified tau fibrils into the tau P301S transgenic mice induced more severe tau pathology and cognitive deficits when compared with tau fibrils. Hence, our results indicate that IAPP cross-seeds tau and mediates the spreading of tau pathology in AD.

## Materials and methods

### Mice

Tau P301S transgenic mice (line PS19) and tau knockout mice were from Jackson Laboratory (stock number: 008169 and 007251, respectively). The mice were housed under standard conditions at 22 °C and a 12 h–12 h light-dark cycle with free access to food and water. Animal care and handling were performed according to the Declaration of Helsinki and approved by the ethical committees of Renmin Hospital, Wuhan University. Three-month-old male mice were used for stereotaxic surgery. All animals were randomly assigned to different groups. The sample size was determined by Power and Precision (Biostat). Investigators were blinded to the group allocation during the animal experiments. The protocol was reviewed and approved by the Animal Care and Use Committee of Renmin Hospital of Wuhan University.

### Human tissue samples

All brain tissues were obtained from the Emory Alzheimer’s Disease Research Center (ADRC) Brain Bank. Human postmortem tissues were acquired under proper Institutional Review Board (IRB) protocols. AD was diagnosed according to the criteria of the Consortium to Establish a Registry for AD and the National Institute on Aging. Diagnoses were further confirmed by the presence of amyloid plaques and neurofibrillary tangles in formalin-fixed tissue. Ages and post-mortem times were similar between AD patients and controls. Informed consent was obtained from the subjects. The study was approved by the Biospecimen Committee.

### ELISA quantification of CSF IAPP concentrations

CSF samples were collected by lumbar puncture from AD patients and age-matched controls in Renmin Hospital of Wuhan University. Informed consent was obtained from the subjects. The study was approved by the ethical committee of Renmin Hospital, Wuhan University. The CSF was centrifuged, aliquoted, and stored at − 80 °C. Quantitative determination of IAPP in CSF was performed using a sandwich ELISA kit (Mlbio, ml022833-C). Briefly, 50 μl of CSF samples were added into each well of the 96-well microtiter plate pre-coated with anti-IAPP antibody and incubated for 1 h at room temperature. After washing the plates five times with PBST (0.05% Tween20 in PBS buffer), 100 μl of HRP-conjugated detection antibody was incubated for 1 h at room temperature. After washing, 100 μl of substrates were added into each well. Signals were measured on a microplate reader (Molecular Devices).

### Real-time RT-PCR

Total mRNA was isolated from AD and control brain hippocampal samples using TRIzol reagent (Invitrogen). The complementary DNA was synthesized using the iScript cDNA synthesis kit (Bio-Rad). Quantitative PCR was performed on a LightCycler 480 real-time PCR system using LightCycler 480 SYBR Green 1 Master Mix (Roche). The following primers were used: IAPP: forward (5′-AGCGGAAATGCAACACTGCCAC-3′) and reverse (5′-CGTTGGTAGATGAGAGAATGGCA-3′); GAPDH: forward (5′-GTCTCCTCTGACTTCAACAGCG-3′) and reverse (5′-ACCACCCTGTTGCTGTAGCCAA-3′). The relative gene expression was normalized to GAPDH expression and assessed using the 2^-ΔΔCt^ method. U2-OS cells were used as the positive control.

### Co-immunoprecipitation

Human AD and control brain tissues were lysed in lysis buffer (50 mM Tris-HCl (pH 7.4), 150 mM NaCl, 1 mM EDTA, 1% Triton x-100, 0.1% SDS, 0.5% sodium deoxycholate) with phosphatase inhibitors (Roche PhosSTOP) and complete protease inhibitor mixture Cocktail (Sigma-Aldrich). The brain lysates were incubated with the IAPP antibody (SAB4501485, MERCK, 1:200) for 1 h at 4 °C. Then 20 μl of Protein A/G PLUS-Agarose (Santa Cruz Biotechnology) were added and incubated for 4 h at 4 °C on a rocker platform. The immunoprecipitates were collected by centrifugation at 1000 rpm for 5 min at 4 °C, and washed 5 times with lysis buffer. The samples were boiled in SDS loading buffer for 5 min and subjected to Western blot analysis using antibodies against IAPP and phosphorylated tau (Thermo, MN1020, 1:1000).

### Preparation of K18 and IAPP pre-formed fibrils (PFFs)

To prepare K18 PFFs, a truncated form of human tau containing only the microtubule-binding domain (K18) was fused with N-terminal His-tag and subcloned into the *Kpn1* site of the *pRK172* bacterial expression vector. The plasmid was transformed into *E. coli BL21 (DE3)* cells. The expression of His-K18 was induced by 1 mM isopropyl β-D-1-thiogalactopyranoside. The cells were sonicated in iced buffer containing 20 mM Tris (pH 8.0), 500 mM NaCl and 5 mM imidazole, and were centrifuged for 30 min at 12,000 r.p.m. at 4 °C. The supernatant was administered to a column pre-equilibrated with Ni-NTA His•Bind Resin (MERCK). The column was washed with 10 column volumes of 20 mM Tris (pH 8.0), 500 mM NaCl and 15 mM imidazole. The protein was then eluted in the same buffer containing 100 mM imidazole. The purified protein was dialyzed against double distilled water for 48 h and then lyophilized. To induce His-K18 fibrillization into PFFs, the lyophilized His-K18 protein was dissolved in PBS with 12.5 μM low-molecular-weight heparin and 2 mM DTT at a final concentration of 1 mg/ml, and were then subsequently incubated for 8–12 h at 37 °C with agitation. To prepare IAPP PFFs, the IAPP and FITC-IAPP peptides were purchased from Qiangyao Biological Technology (Shanghai). IAPP was first dissolved at a concentration of 1.0 mg/ml in 2 mM HCl (pH 2.0), and then diluted to a final concentration of 0.01 mg/ml and 0.05 mg/ml in PBS, respectively. The samples were incubated at room temperature with shaking (450 r.p.m.) for 90 min. Thioflavin T (ThT) fluorescence assay was conducted to assess the aggregation process of K18 and IAPP. Briefly, the samples were incubated with 20 μM ThT, and read at excitation and emission wavelengths of 440 nm and 485 nm, respectively, with a slit width of 5 nm on a microplate reader (Molecular Device).

### Transmission electron microscopy (TEM)

PFFs formed by K18 in the presence or absence of IAPP were visualized by negative stain TEM as described previously [24]. Tau PFFs and IAPP-tau PFFs were adsorbed to glow discharged 400 meshed carbon-coated copper grids for 2 min, quickly washed three times with Tris-HCl buffer (50 mM, pH 7.4), and floated upon two drops of 0.75% uranyl formate for 30 s. The grids were allowed to dry before imaging on a Phillips CM 120 TEM operating at 80 kV. The images were captured and digitized with an ER-80 CCD (8 megapixel) by advanced microscopy techniques.

### Immune-electron microscopy (immune-EM)

For immunogold labeling, 10 μL of the IAPP-tau fibrils were added to Formvar/carbon-coated 200 mesh copper TEM support grids (EMS) and blocked with normal goat serum. After glutaraldehyde fixation, the grid was incubated with a mixture of primary antibodies (mouse anti-His and rabbit anti-IAPP). After rinsing, the grid was incubated in a mixture of secondary colloidal gold antibodies-4 nm goat anti-mouse IgG and 8 nm goat anti-rabbit IgG. 2% uranyl acetate solution was placed on the grid, rinsed, and allowed to air-dry. Grids were imaged in a JEOL 1200 transmission electron microscope at 80 kV, and images were captured with a 2 × 2 k Gatan Orius CCD camera.

### GST pull-down assay

Plasmids encoding GST-vector or GST-IAPP were transfected into HEK293 cells stably expressing GFP-tau 2N4R or YFP-tau four microtubule binding repeat domain (tau-RD). Forty-eight hours later, the cell lysates were incubated with Glutathione agarose beads overnight at 4 °C. The beads were then washed 4 times in PBS with 0.1% Triton X-100 and boiled in SDS loading buffer. The binding between IAPP and tau was then measured by Western blot analysis.

### Preparation of 1% Triton X-100-soluble and 2% SDS-soluble protein extraction

Mouse tissue was mechanically homogenized in buffered-Triton X-100 (20 mM Tris–HCl, 140 mM NaCl, 1% Triton X-100, pH 7.4) containing protease and phosphatase inhibitors. The homogenates were ultracentrifuged (4 °C for 60 min) at 100,000×g (Beckman Coulter). The supernatants were aliquoted and stored at − 80 °C. The pellets were further extracted in buffered-SDS (2% SDS in 20 mM Tris–HCl, pH 7.4, 140 mM NaCl), centrifuged as above, and the supernatants were stored at − 80 °C.

### Western blot

The mouse brain tissue or cultured cells were lysed for 30 min at 4 °C in lysis buffer (50 mM Tris, pH 7.4, 40 mM NaCl, 1 mM EDTA, 1% Triton X-100, 0.1% sodium dodecyl sulfate (SDS), 50 mM NaF, 10 mM sodium pyrophosphate, and 10 mM sodium β-glycerophosphate, supplemented with protease inhibitors cocktail), and centrifuged for 30 min at 15,000 r.p.m. The protein concentration of the supernatant was determined by Pierce BCA Protein Assay Kit (Thermo). After protein separation by 8–12% SDS-PAGE, the samples were transferred to a nitrocellulose membrane. The membranes were blocked with 5% non-fat milk in TBS containing 0.1% Tween 20 (TBST), and were then incubated with primary antibodies overnight at 4 °C. The membranes were washed 3 times in TBST and incubated with horseradish peroxidase (HRP)-conjugated anti-mouse or anti-rabbit antibodies for 1 h at room temperature. Immunoreactivity was visualized by enhanced chemiluminescence (ECL) using ECL Western blotting system (Bio-Rad). Primary antibodies to the following targets were used: GST (Proteintech, 66,001–2-Ig, 1:5000), EGFP-HRP (Proteintech, HRP-66002, 1:5000), His (Proteintech, 66,005–1-Ig, 1:5000), GAPDH (Proteintech, 60,004–1-Ig, 1:5000), AT8 (Thermo, MN1020, 1:1000), AT100 (Thermo, MN1060, 1:1000), tau5 (Thermo, MA5–12808,1:1000), IBA1 (Proteintech, 10,904–1-AP, 1:800), GFAP (Proteintech, 60,190–1-Ig, 1:1000), PSD 95 (Cell Signaling Technology, 3409 s, 1:1000), synapsin I (Cell Signaling Technology, 2312 s, 1:1000), and synaptophysin (Cell Signaling Technology, 36,406 s, 1:1000).

### Immunohistochemistry and immunofluorescence

Mice were anaesthetized and then were transcardially perfused with cold PBS and 4% paraformaldehyde (PFA). The brains were stored for 24 h in 4% PFA at 4 °C, and then embedded in paraffin. Serial 5-μm-thick sections from all animal groups were processed in parallel for immunohistochemistry and immunofluorescence. For immunofluorescent staining of human brain tissue, the brain sections were first immersed in 0.1% Sudan Black B (SSB) and 70% ethanol for 20 min at room temperature to eliminate the autofluorescence signal. Then, the slides were washed three times with 0.02% Tween 20 in PBS to wash out SBB. After blocking the endogenous peroxidase activity with 3% H_2_O_2_ for 10 min and washed 3 times in PBS, sections were blocked in PBS with 1% BSA and 0.3% Triton X-100 for 30 min followed by overnight incubation with the primary antibodies at 4 °C. Sections were washed three times in PBS with 0.1% Triton X-100. For immunohistochemistry, the signal was developed with a Histostain-SP kit (Invitrogen). For immunofluorescent staining, the slides were incubated with secondary antibodies conjugated to Alexa Fluor 488 or Alexa Fluor 594. Primary antibodies to the following targets were used: MAP 2 (Proteintech, 17,490–1-AP, 1:1000), AT8 (Thermo, MN1020, 1:1000), AT100 (Thermo, MN1060, 1:1000), IBA1 (Wako, 019–19,741,1:1000), GFAP (Proteintech, 60,190–1-Ig, 1:1000), p-Tau Ser396 (Thermo, 710,298, 1:1000), IAPP (EMD Millipore, ABN1456, 1:100).

### Primary neuronal culture

Primary cortical neurons were derived from tau P301S mice and tau knockout mice at embryonic day 18, and cultured in the Neurobasal medium supplemented with B27. IAPP PFFs, Tau PFFs, or IAPP-tau PFFs were added to the culture medium (5 μg/ml) at 6 days in vitro (DIV). Five days later, the neurons were first fixed and permeabilized with 4% paraformaldehyde containing 1% Triton X-100 for 10 min to eliminate the soluble tau, and then immunostained with AT8, AT100, p-tau Ser396, and p-tau Ser202 antibodies, respectively. The sections were examined under a fluorescence microscope (Olympus). The neurotoxic effect of the PFFs was determined by the TUNEL BrightRed Apoptosis Detection Kit (Vazyme) and Hoechst/PI staining.

### Stereotaxic surgery

Three-month-old mice were anaesthetized and placed into an automated stereotaxic instrument. The mice received a unilateral stereotaxic injection of PBS, IAPP PFFs, tau PFFs, or IAPP-tau PFFs using Hamilton syringes into the hippocampus at AP-2.5 mm, L-2.0 mm, DV-1.8 mm. The PFFs (1 μg/μl) were prepared before use. Each mouse received 5 μl PFFs at a speed of 0.2 μl per min. Mice were monitored until they were fully recovered. To test the transfer of circulating IAPP into the brain, 100 μg of FITC-tagged IAPP PFFs were injected into the wild-type mice and tau P301S mice through the tail vein. The presence of FITC-IAPP PFFs in the brain was tested 24 h later.

### Morris water maze test

Morris water maze test was used to detect the spatial learning and memory of the tau P301S mice as described previously [25]. Mice were trained for four trials per day for 7 consecutive days. The mice were allowed to search for the platform for 60 s. Otherwise, they would be guided to the platform manually, and were allowed to stay on it for 15 s before the next trial. After the trials, mice were dried and put back to the cage. After the last training day (day 7), a spatial probe trial was performed. The platform was removed and the mice were allowed to swim for 60 s. The percentage of time spent in the platform quadrant was measured. All trials were recorded by a computerized tracking system that analyzed the distance the mice moved, latency required to reach the platform, and swim speed using ANY-Maze software (San Diego Instruments).

### Y-maze test

Three arms were randomly designated at a 120 degrees angle from each other, including a start arm, a novel arm, and the third arm. On the first trial (training), mice were allowed to explore only the start arm and the third arm for 5 min with the novel arm being blocked. After 1 h inter-trial interval, the second trial (retention) was conducted and the mice had free access to all three arms for 5 min. The number of entries and time spent in the new arm were recorded and analyzed with the ANY-Maze software.

### Electron microscopy of synapses

Synaptic structure and density were determined by electron microscopy. Mice were anesthetized and perfused transcardially with 2% glutaraldehyde. Then the hippocampal slices were postfixed in 1% cold OsO4 for 1 h. Samples were prepared and examined using standard procedures. Ultrathin sections (90 nm) were stained with uranyl acetate and lead acetate and viewed at 100 kV in a JEOL 200CX electron microscope. Synapses were identified by the presence of synaptic vesicles and postsynaptic densities. Synapse density, the number of synaptic clefts, synaptic active zone length, and width of the synaptic clefts in the CA1 area of the hippocampus were calculated.

### Electrophysiology

All electrophysiological recordings were performed using a whole-cell patch-clamp. Briefly, mice were deeply anesthetized. When all pedal reflexes were abolished, brains were removed and dropped in an ice-cold oxygenated cutting solution containing the following (in mM): 25 D-glucose, 2.5 KCl, 1.26 NaH_2_PO_4_, 25 NaHCO_3_, 7.2 MgCl_2_, 0.5 CaCl_2_, 3.1 Na-pyruvate, 11.35 ascorbic acid and 97 choline chloride. Coronal slices (350-μm thick) containing the dorsal hippocampus were cut at 4 °C in the cutting solution using a Leica VT1200S vibratome and then transferred to an incubation chamber filled with oxygenated artificial cerebrospinal fluid (aCSF), which contains the following: 118 mM NaCl, 2.5 mM KCl, 1 mM NaH_2_PO_4_, 26 mM NaHCO_3_, 2 mM MgCl_2_, 2 mM CaCl_2_, and 22 mM glucose in a 35 °C water bath for 30 min and then put in room temperature for 30 min before being recorded. The brain slices were transferred to a chamber perfused with the different recording bath solutions. Cells were approached under DIC with ~ 6 MΩ pipettes pulled from borosilicate glass (Warner Instruments, Inc) using a horizontal drawing puller (Sutter Instrument), and impaled until forming high resistance seal (GΩ) between the recording pipette and the cell membrane. Then, the whole-cell configuration was established by the gentle application of negative pressure through the recording pipette. The recording pipette contained: 100 mM CsCH_3_SO_3_, 20 mM KCl, 10 HEPES, 4 mM Mg-ATP, 0.3 mM Tris-GTP, 7 mM Tris2-Phosphocreatine, 3 mM QX-314, osmolarity: 298 mOsm; pH 7.3 adjusted with KOH. Electrical signals were recorded at 25 kHz with Axon clamp 700B amplifier (Axon Instruments), digitalized with a Digidata 1440 digitizer (Molecular devices) that was in turn controlled by Clampex 10.1 (Molecular Devices).

### Golgi staining

Mouse brains were fixed in 10% formalin for 24 h and then immersed in 3% potassium bichromate for 3 days in the dark. The solution was changed every day. Then the brains were transferred into 2% silver nitrate solution and incubated for 24 h in the dark. Vibratome sections were cut at 60 μm, air-dried for 10 min, dehydrated through 95 and 100% ethanol, cleared in xylene, and assembled on coverslips. Bright-field images of pyramidal neurons in the hippocampus and cortex were taken at 100 × magnification using a Zeiss Axioplan (Zeiss, Decatur, GA, USA) microscope. To measure the spine density, all clearly evaluable areas of 50–100 μm of secondary dendrites from each imaged neuron were counted.

### Statistical analysis

Statistical analysis was performed with GraphPad Prism 7.0 (GraphPad Prism, San Diego, CA, USA). Independent data were introduced into the statistical analysis [26]. The data are shown as means ± SEM. Statistical comparisons between two groups were performed with Student’s *t*-tests. For analysis of more than two groups, one-way ANOVA test was performed followed by post hoc analysis where appropriate. Differences with *P* values less than 0.05 were considered significant.

## Results

### IAPP interacts with tau in human AD brain

**Fig. 1:**
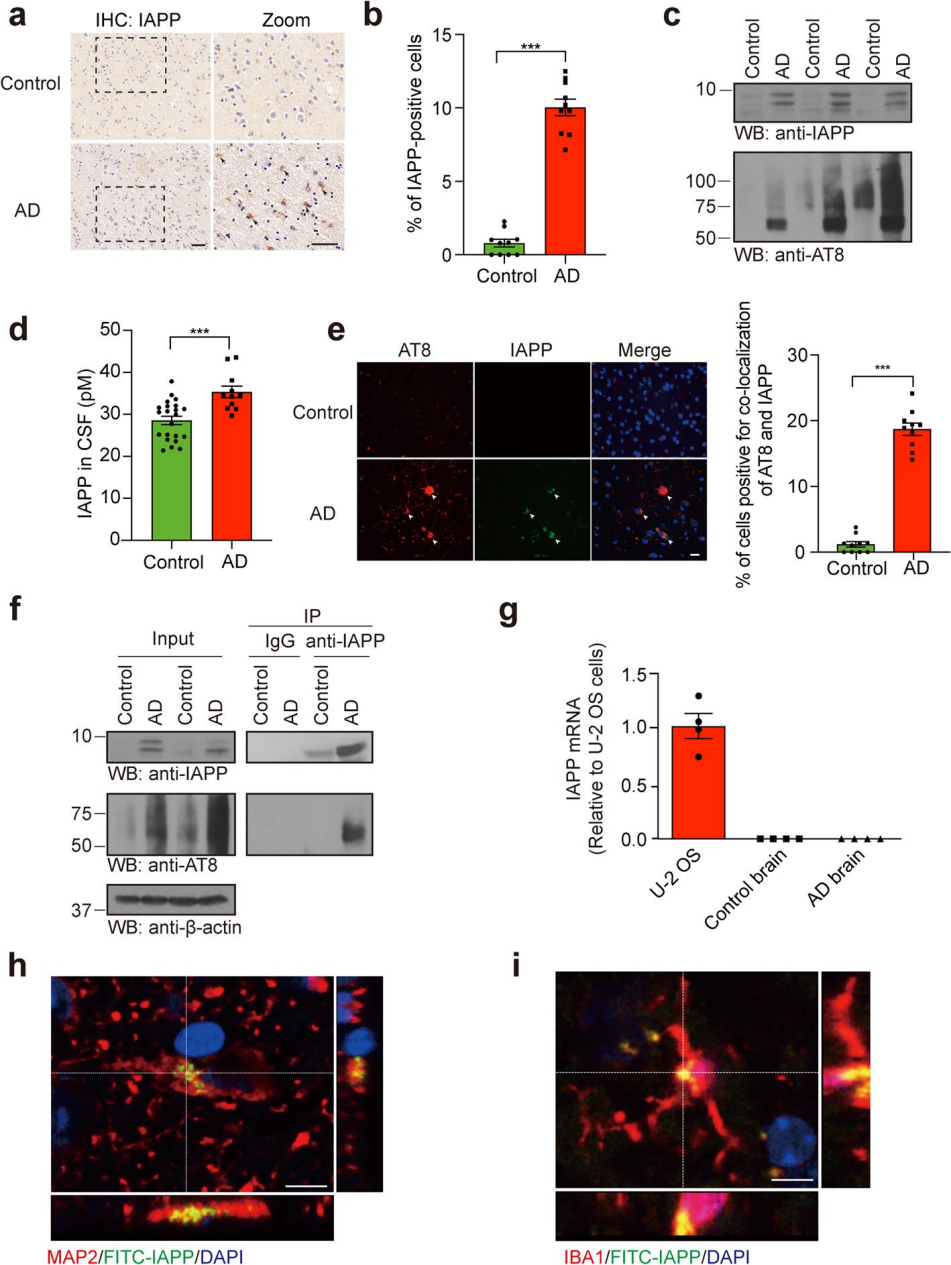
IAPP deposits in the brain of AD patients. **a** Representative images of IAPP deposition in the hippocampal sections of AD patients and age-matched controls. **b** Quantification of the percentage of IAPP-positive cells. Scale bars, 50 μm. Bars represent means ± SEM. Unpaired Student’s *t-*test. *n* = 10 slides from 10 AD patients and 10 slides from 10 control subjects. ***P < 0.001. **c** Western bolt assay detects the presence of IAPP in the formic acid fraction from AD brains. *n* = 3 AD patients and 3 controls. **d** Concentrations of IAPP in the CSF of AD patients (*n* = 11) and healthy controls (*n* = 22). Bars represent means ± SEM. Unpaired Student’s *t*-test, ***P < 0.001. **e** Co-immunostainings of IAPP and phosphorylated tau (p-tau) in brain sections of AD patients. *n* = 10 AD patients and 10 controls. Scale bars, 20 μm. **f** Co-immunoprecipitation showing the interaction between IAPP and p-tau in AD human brains. *n* = 3 AD patients and 3 controls. **g** RT-PCR analysis found no IAPP mRNA expression in both AD and control brains. *n* = 4 AD patients and 4 controls. **(h, i)** Uptake of intravenously injected pure IAPP PFFs by neurons (**g**) and microglia (**h**) in tau P301S mice. Scale bars, 20 μm

To examine whether IAPP participates in tau pathology in AD, we immunostained IAPP in hippocampal brain slides from AD patients and age-matched controls. We found abundant IAPP signals in AD brains, which were hardly detected in age-matched control brains (Fig. 1a, b, Table S1). Western blot analysis confirmed the presence of IAPP in the formic acid fraction from the AD brain (Fig. 1c). Furthermore, we performed a sensitive enzyme-linked immunosorbent assay (ELISA) to determine the concentration of IAPP in the CSF from control subjects and AD patients (Table S2), and found that the concentrations of IAPP in the CSF of AD patients were much higher than that in the healthy controls (Fig. 1d). We further co-stained IAPP with neuronal and glial markers, and found that IAPP was localized within neurons (MAP2) and microglia (IBA1), but not in astrocytes (GFAP) (Fig. S1a). Lastly, immunofluorescence staining indicated that IAPP co-localized with phosphorylated tau in the human AD brain slides (Fig. 1e, Fig. S1b). To determine whether IAPP directly binds phosphorylated tau in vivo, we performed co-immunoprecipitation from human tissues and confirmed the interaction between IAPP and phosphorylated tau in AD brains (Fig. 1f). We further performed GST pull-down assay in HEK293 cells co-transfected with GST-tagged IAPP and GFP-tau 2N4R. We found that IAPP binds tau (Fig. S1c). A truncation assay revealed that tau RD is sufficient to associate with IAPP (Fig. S1d). To investigate the source of IAPP in the AD brain, we performed RT-PCR and found no IAPP mRNA expression in control and AD brains (Fig. 1g), suggesting the IAPP detected in the brain is from the peripheral organs. To further assess whether circulating IAPP can enter the brain, we injected FITC-labeled pure IAPP PFFs into 10-month-old wild-type mice and tau P301S mice through the tail vein. Fluorescence was detected to be localized within both neurons and microglia in the mice brain 24 h post-injection (Fig. 1h, i, Fig. S1e-h), indicating that peripheral IAPP PFFs can pass the BBB and be taken up by neurons and microglia. Furthermore, more fluorescence signals were detected in tau P301S mice than wild-type mice (Fig. S1g, h), indicating that overexpression of tau P301S facilitates the trafficking of circulating IAPP into the brain.

### IAPP promotes the formation of more toxic tau PFFs

**Fig. 2:**
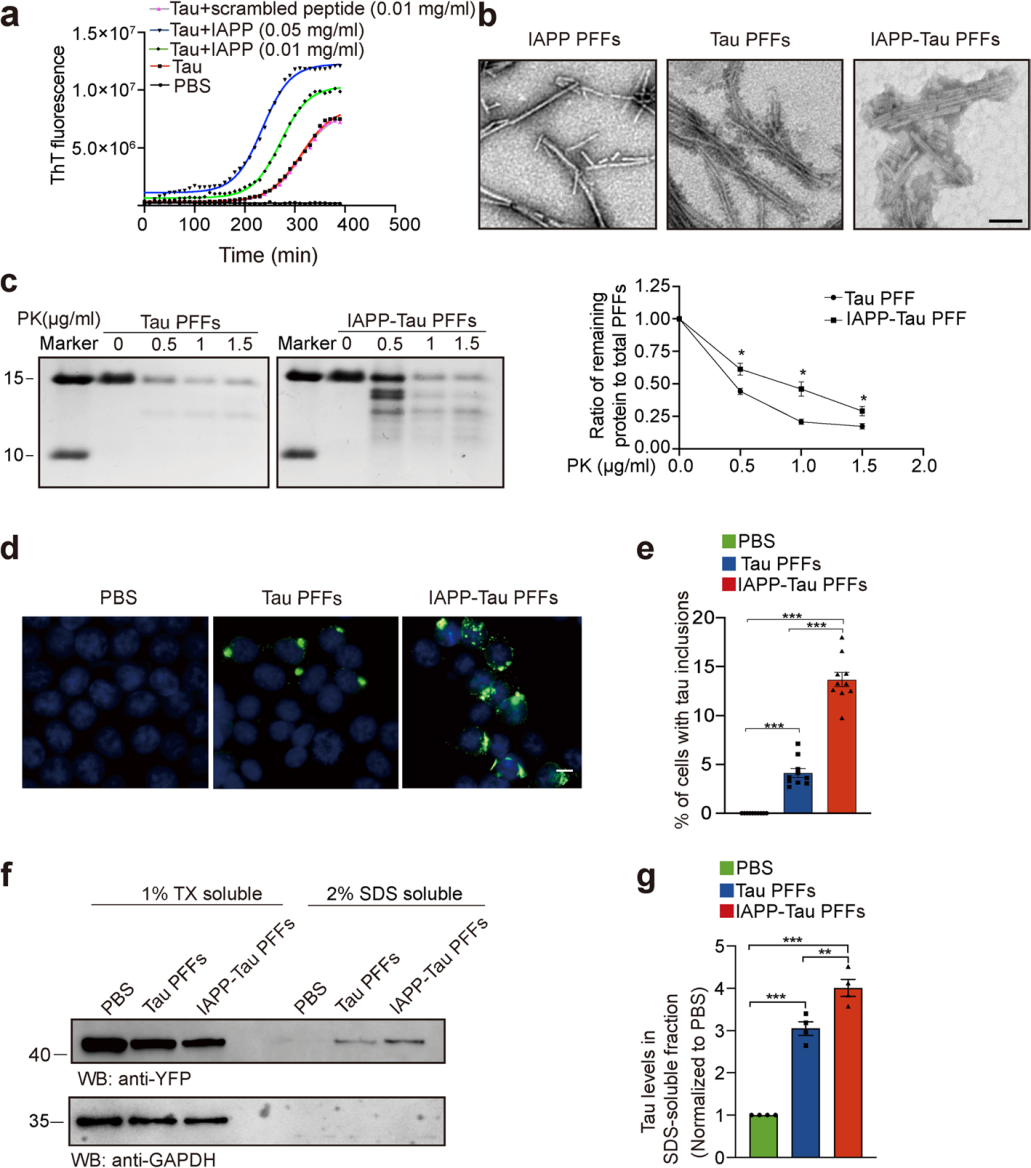
IAPP accelerates tau fibrillization in vitro. **a** ThT fluorescence assay of tau fibrillization in the presence of different concentrations of IAPP monomers or scrambled peptide (*n* = 3 independent experiments. The mean ThT fluorescence are shown). **b** Representative images of transmission electron microscopy (TEM) for IAPP PFFs, Tau PFFs, and IAPP-tau PFFs. Scale bar, 200 nm. **c** PK digestion assay. Tau PFFs or IAPP-tau PFFs were incubated with increasing concentrations of PK (0 to 1.5 mg/ml) and analyzed by Coomassie blue staining (left). Quantification represents the ratio of remaining protein to total PFFs (right). Data are means ± SEM (*n* = 3 independent experiments). **d** Tau PFFs and IAPP-tau PFFs induce tau aggregation in HEK293 cells stably expressing YFP-tagged tau RD. Scale bars, 20 μm. **e** The percentage of cells with tau inclusions induced by tau PFFs and IAPP-tau PFFs. Data are presented as means ± SEM. One-way ANOVA followed by Tukey’s post hoc test (*n* = 10 slices from 3 independent experiments). ***P < 0.001. **f, g** Cells transduced with tau PFFs and IAPP-tau PFFs were sequentially extracted with 1% Triton X-100 (TX-100) (TX soluble) and 2% SDS (TX insoluble). Lysates were subjected to Western blot to show the presence of tau in different fractions (*n* = 4 independent samples). Data are presented as means ± SEM. One-way ANOVA followed by Tukey’s post hoc test. **P < 0.01, ***P < 0.001. DAPI, 4′,6-diamidino-2-phenylindole

IAPP is an intrinsically disordered peptide that is prone to aggregate [27]. We thus determined whether IAPP influences tau aggregation. ThT fluorescence assay found that IAPP accelerated the aggregation of K18 in a concentration-dependent manner. The fibrilization process of K18 (1 mg/ml) was markedly accelerated in the presence of IAPP monomer (0.01 mg/ml and 0.05 mg/ml) (Fig. 2a), or IAPP PFFs (0.01 mg/ml) (Fig. S2a). However, a scrambled peptide failed to accelerate the fibrilization process of K18 (Fig. 2a). To determine whether IAPP alter the properties of tau aggregates, we performed a series of biochemical analysis using tau PFFs and IAPP-modified tau PFFs. First, morphological analysis using electron microscopy showed that the IAPP-tau PFFs are more compact than the tau PPFs (Fig. 2b). Immuno-electron microscopy of IAPP-modified tau PFFs found that IAPP was incorporated into tau filaments (Fig. S2b). Next, we performed proteinase K (PK) digestion assay using tau PFFs and IAPP-tau mixed PFFs. The digestion products of the tau PFFs predominantly consist of a band at about 12 kDa. Notably, digestion of the IAPP-tau PFFs generated several novel bands, which correspond to a different digestion pattern of tau or some form of IAPP aggregates. Furthermore, the ratio of remaining fragments is much higher in the IAPP-tau PFFs than the tau PFFs (Fig. 2c). These results indicate that IAPP induces the formation of IAPP-tau mixed PFFs with distinct biochemical characteristics.

We further compared the seeding activity of tau PFFs and IAPP-tau PFFs by transfecting the same amount of PFFs into HEK293 cells stably transfected with YFP-tau RD (Fig. S2c-f). Both tau PFFs and IAPP-tau PFFs induced tau aggregation, with much more aggregation induced by the IAPP-tau PFFs (Fig. 2d, e). Furthermore, the IAPP-tau PFFs induced much abundant tau species in the triton-insoluble fraction (Fig. 2f, g), indicating that IAPP enhances the seeding activity of tau fibrils.

### IAPP-tau PFFs induce tau phosphorylation and neurotoxicity in vitro

**Fig. 3:**
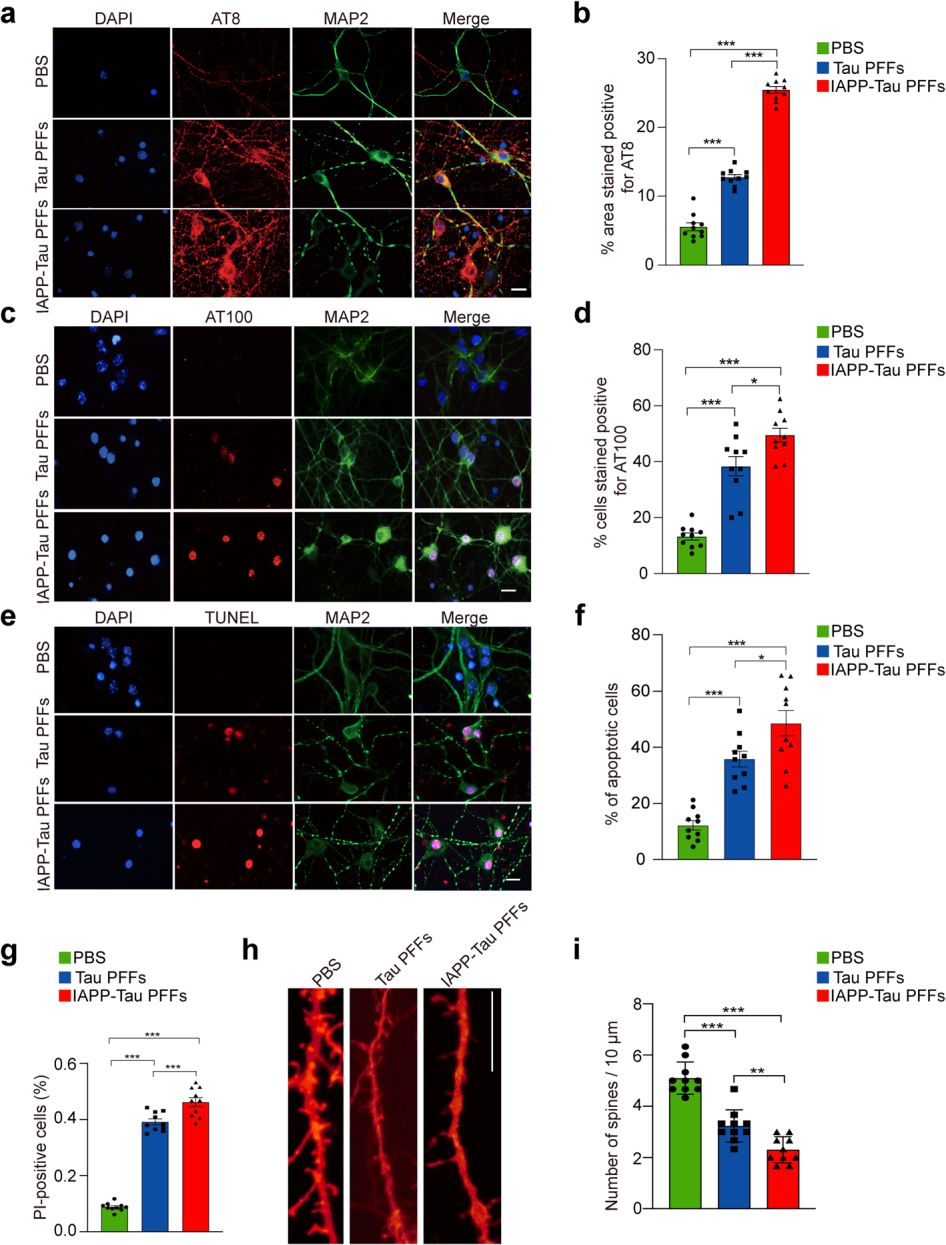
IAPP-tau PFFs induce tau phosphorylation and neuronal apoptosis in vitro. **a-d** Representative immunostaining and quantification of AT8 (**a, b**) and AT100 p-tau (**c, d**) in primary neurons transduced with tau PFFs or IAPP-tau PFFs. Scale bar, 20 μm. **e** Representative images of TUNEL staining of primary cortical neurons transduced with tau PFFs or IAPP-tau PFFs. **f, g** Quantification of TUNEL staining (**f**) and Hoechst/PI staining (**g**). Scale bars, 30 μm. **h, i** Representative images and quantification of Dil staining of dendritic spines in neurons. Experiments were independently performed three times. Fifty visual fields (Fig. 3b, d), 150 cells (Fig. 3f, g), and 50 dendrites (Fig. 3i) from 10 slices were counted in each group. Scale bar, 20 μm. Bars represent means ± SEM. One-way ANOVA followed by Tukey’s post hoc test (*n* = 10 slices). *P < 0.05, **P < 0.01, ***P < 0.001

To investigate the effect of IAPP PFFs on tau phosphorylation and neurotoxicity, we transduced IAPP PFFs into primary cultured neurons, and found that IAPP PFFs induced tau phosphorylation and neuronal apoptosis (Fig. S3a-d). To assess whether tau is required for IAPP-induced neurodegeneration, we cultured primary neurons from tau knockout mice, and then treated the neurons with IAPP PFFs. Interestingly, the deletion of tau abolished the detrimental effect of IAPP PFFs (Fig. S3e, f). We then compared the effect of tau PFFs and IAPP-tau PFFs on tau phosphorylation. The neurons were transduced with PBS, tau PFFs or IAPP-tau PFFs, respectively. IAPP-tau PFFs induced much higher levels of tau phosphorylation at different sites (Ser202/Thr205, Thr212/Ser214, Ser396) when compared with tau PFFs (Fig. 3a-d, Fig. S4a-d). These results were verified by Western blot analysis (Fig. S4e, f). We further tested the neurotoxic effect of tau PFFs and IAPP-tau PFFs on primary neurons. TUNEL assay found that IAPP-tau PFFs induced a much higher apoptotic rate than the tau PFFs (Fig. 3e, f), indicating that IAPP enhances the toxicity of tau PFFs. Similar results were found by Hoechst/PI staining (Fig. 3g). Finally, we performed Dil staining to illustrate the density of dendritic spines in neurons, and found that IAPP-tau PFFs induced more severe degeneration of the dendritic spines (Fig. 3h, i). Together, these results indicate that IAPP-tau PFFs are more potent in inducing tau phosphorylation and neuronal toxicity.

### IAPP PFFs promote tau pathology and cognitive decline in vivo

To detect the effect of IAPP PFFs on tau pathology in vivo*,* we injected IAPP PFFs into the hippocampus of tau P301S transgenic mice. One month after injection, more abundant tau phosphorylation was observed in mice injected with IAPP PFFs when compared with PBS-injected mice in the ipsilateral hippocampus, dentate gyrus and entorhinal cortex, and contralateral hippocampus. Three months after injection, the difference was further extended to the contralateral dentate gyrus and cortex (Fig. S5a-e). We also investigated the effect of IAPP PFFs on cognitive impairments using the Morris water maze test and Y-maze test. The result showed significant cognitive decline in mice injected with IAPP PFFs compared to mice injected with PBS (Fig. S5f-j). The expression of synaptic markers including synaptophysin, synapsin I, and PSD95 was decreased likewise (Fig. S5k, l). These results indicate that IAPP PFFs induce tau phosphorylation and cognitive decline in vivo.

### IAPP-tau PFFs promote the propagation of tau pathology in vivo

**Fig. 4:**
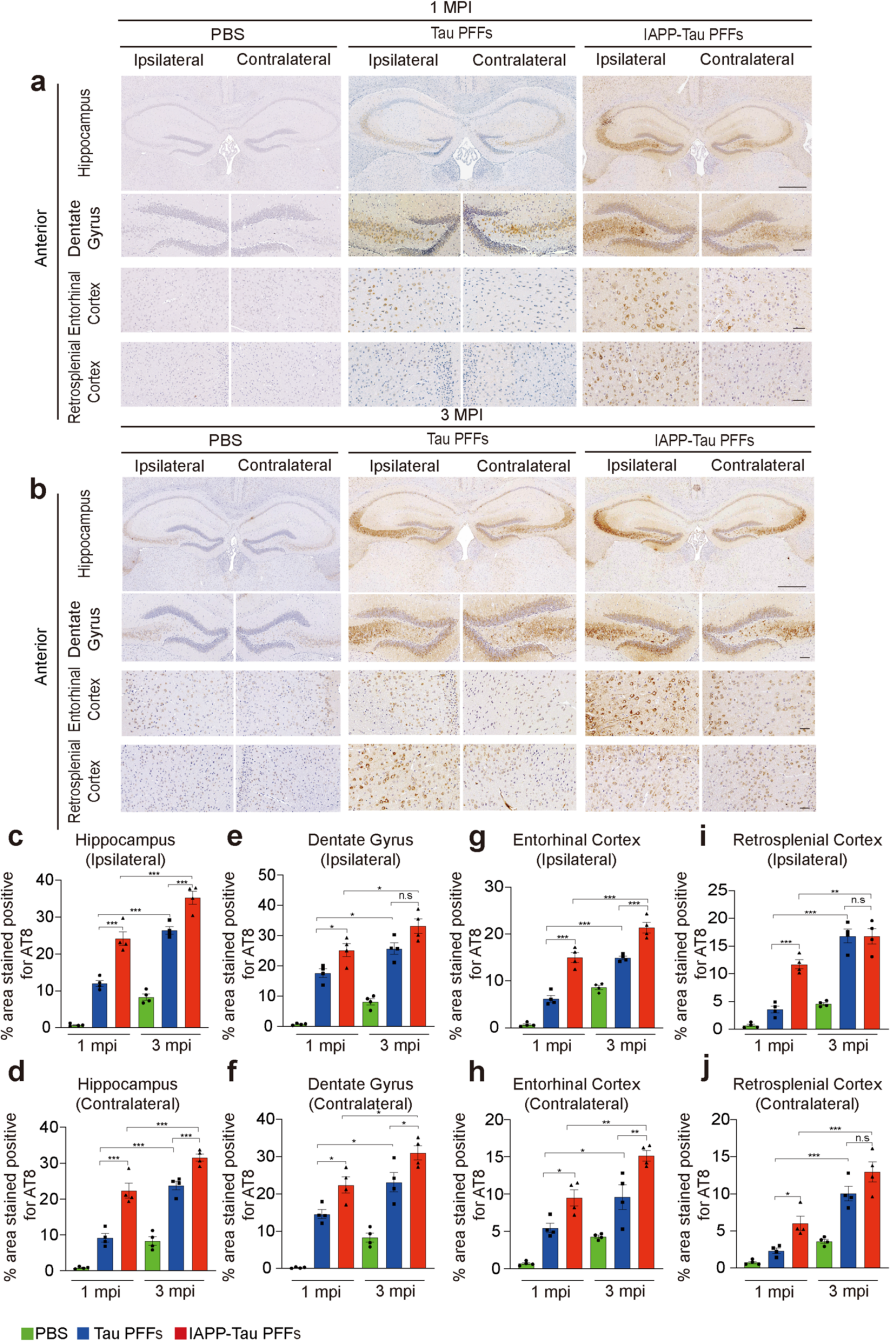
IAPP-Tau PFFs promote the propagation of tau pathology in vivo. **a, b** Representative images of AT8 p-tau pathology in tissue sections (anterior hippocampal level) from the hippocampus, dentate gyrus, retrosplenial cortex, and entorhinal cortex of tau P301S mice 1 month (**a**) and 3 months (**b**) after the injection of PBS, tau PFFs or IAPP-tau PFFs. Scale bar, 200 μm in (**a**) and (**b**) upper panel, 50 μm in (**a**) and (**b**) lower panels. **c-j** Quantification of p-tau pathology of ipsilateral and contralateral sides in the hippocampus (**c, d**), dentate gyrus (**e, f**), entorhinal cortex (**g, h**), and retrosplenial cortex (**i, j**). *n* = 4 mice per group. Data are presented as means ± SEM. One-way ANOVA followed by Tukey’s post hoc test. *P < 0.05, **P < 0.01, ***P < 0.001, n.s. not significant

To determine the effect of IAPP-tau PFFs on tau pathology in vivo, we injected tau PPFs or IAPP-tau PFFs into the hippocampus of tau P301S transgenic mice. The PFFs dose in different groups was unified before injection (Fig. S2c, d). The propagation of tau pathology was determined 1 month and 3 months post-injection (MPI) at the anterior and posterior hippocampus levels, respectively. One month after injection, both tau PFFs and IAPP-tau PFFs induced the phosphorylation of tau in the injection site and the spreading of tau pathology to the structurally connected brain areas, including the dentate gyrus, entorhinal cortex, and retrosplenial cortex (Fig. 4a, Fig. S6a). The mice injected with IAPP-tau PFFs showed increased tau phosphorylation when compared with those injected with tau PFFs. The effect was observed in brain areas both ipsilateral and contralateral to the injection site. Three months after injection, more abundant tau phosphorylation was observed in both PFFs groups. The IAPP-tau PFFs group showed much more severe phosphorylation of tau when compared with the tau PFFs group in the ipsilateral hippocampus and entorhinal cortex, and contralateral hippocampus, dentate gyrus, and entorhinal cortex (Fig. 4b-j, Fig. S6b-l). The extent of tau phosphorylation in the ipsilateral dentate gyrus and retrosplenial cortex, and contralateral retrosplenial cortex was not statistically different between mice injected with tau PFFs and IAPP-tau PFFs, suggesting the phosphorylation of tau was saturated in these areas in both groups (Fig. 4e, i, j). Furthermore, we compared the degree of pathological changes in tau P301S mice injected with PBS, IAPP PFFs, tau PFFs and IAPP-tau PFFs. The IAPP-tau PFFs showed the most severe tau pathology, followed by tau PFFs and IAPP PFFs (Fig. S7a-d). Overall, these results indicate that the IAPP-tau PFFs facilitate the propagation of tau pathology in vivo.

### IAPP-tau PFFs promote tau phosphorylation and neuroinflammation in vivo

**Fig. 5:**
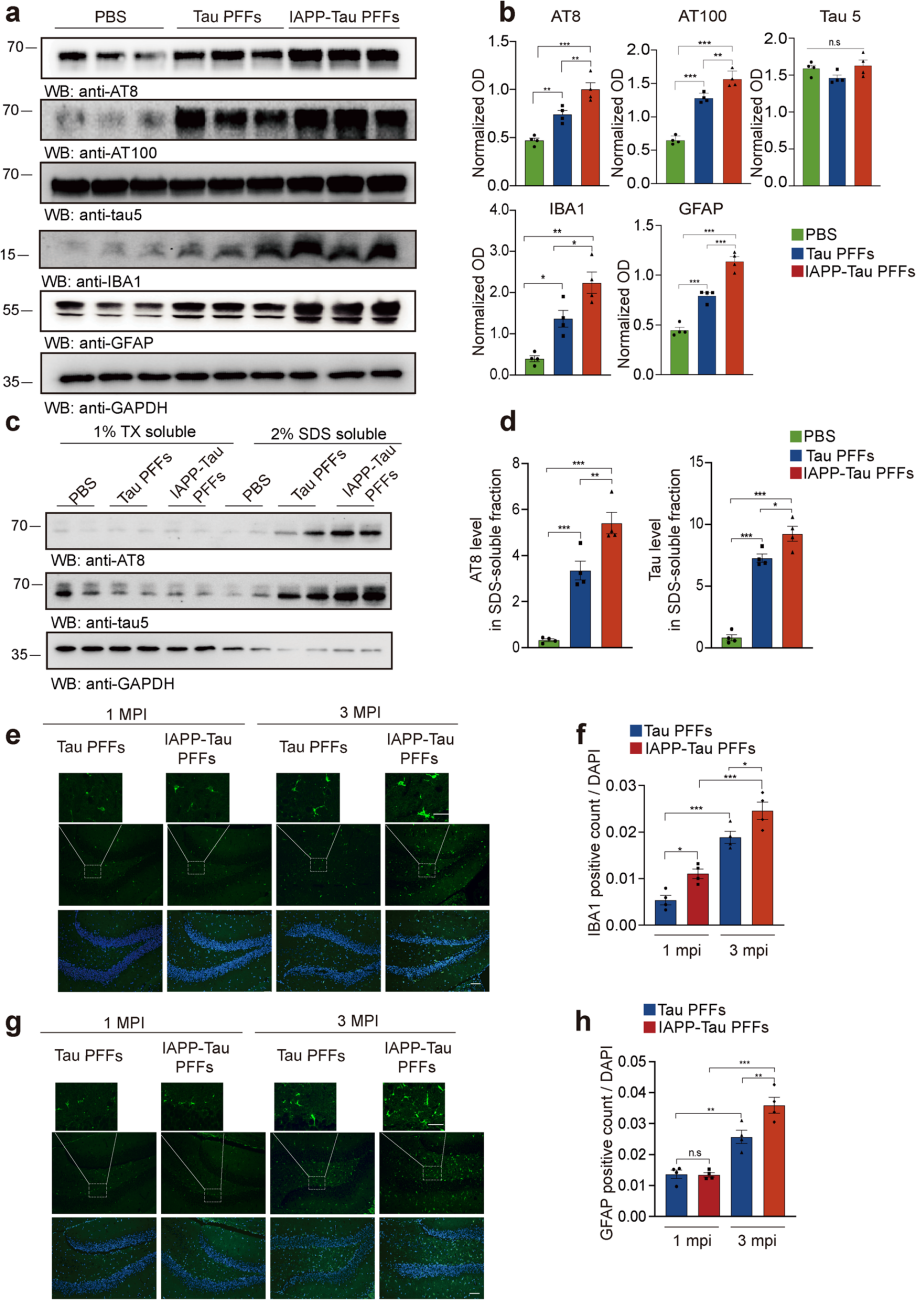
IAPP-Tau PFFs promote tau phosphorylation and neuroinflammation in vivo. **a** Representative immunoblots of p-tau (AT8 and AT100), total-tau (tau5), IBA-1, and GFAP in the ipsilateral hippocampus of mice injected with PBS, tau PFFs or IAPP-tau PFFs. **b** Statistical analysis shows significantly increased expression of AT8, AT100, IBA1, and GFAP in mice injected with IAPP-Tau PFFs, compared to mice injected with PBS and tau PFFs. **c, d** Ipsilateral hippocampal samples were sequentially extracted with 1% Triton X-100 (TX-soluble) and 2% SDS (TX-insoluble) from mice injected with either PBS, tau PFFs, or IAPP-tau PFFs. Quantification is presented in (**d**). **e-h** Immunostaining and quantification of microglia marker IBA1 (**e, f**) and astrocyte marker GFAP (**g, h**) in mice 3 months after injection with tau PFFs or IAPP-tau PFFs. Scale bar, 50 μm for lower panel, 20 μm for magnification. *n* = 4 mice per group. Data are presented as means ± SEM. One-way ANOVA followed by Tukey’s post hoc test. *P < 0.05, **P < 0.01, ***P < 0.001

We further investigated the phosphorylation of tau in mice brains injected with tau PFFs and IAPP-tau PFFs. Western blot analysis showed a significant increase of p-tau, but not total-tau in both PFFs groups compared to the PBS-injected mice. The IAPP-tau PFFs induced more abundant tau phosphorylation in the ipsilateral hippocampus in contrast to tau PFFs (Fig. 5a, b). A similar pattern of tau phosphorylation was also found in the ipsilateral cortex (Fig. S8a, b). Insoluble tau species were found in mice injected with tau PFFs. IAPP-tau PFFs substantially increased the content of insoluble tau fraction (Fig. 5c, d, Fig. S8c, d). Neuroinflammation contributes to neurodegeneration in AD. Thus, we further investigated the effect of IAPP-tau PFFs on the activation of microglia and astrocytes. We found that microglia and astrocytes were highly activated in the IAPP-tau PFFs group at both 1 month and 3 months after injection, as demonstrated by the expression of microglia marker IBA1 and astrocyte marker GFAP in the brain lysates (Fig. 5a, b), as well as the presence of IBA-1- and GFAP-positive cells in the brain slides (Fig. 5e-h, Fig. S9a-d).

### IAPP-tau PFFs induce synaptic dysfunction and memory deficits in tau P301S mice

**Fig. 6:**
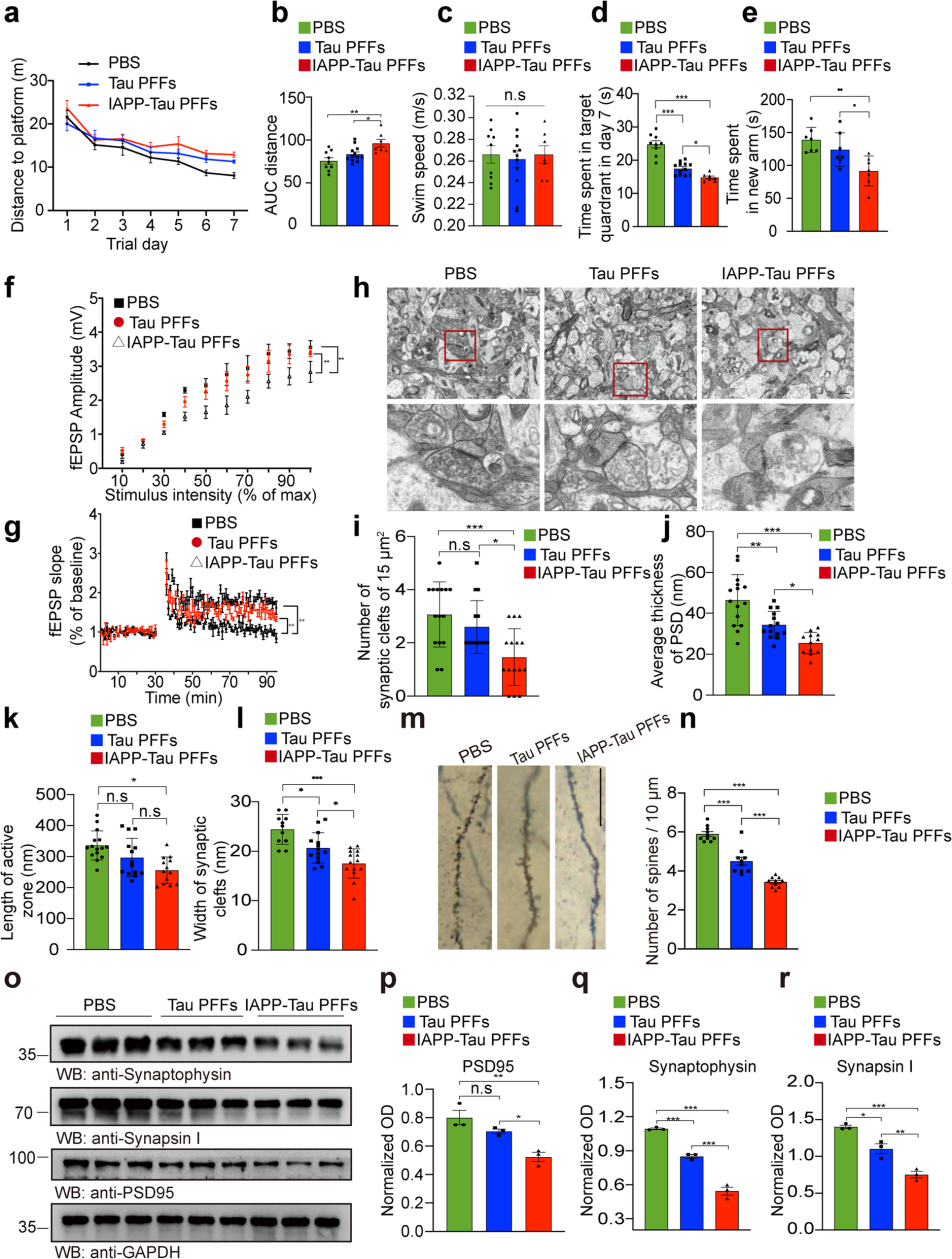
IAPP-tau PFFs induce cognitive impairment and synaptic dysfunction in vivo. Three-month-old male mice were injected with PBS, tau PFFs or IAPP-tau PFFs, respectively. The mice were sacrificed three months after PFF injection. **a** Spatial memory was assessed by the Morris water maze test. Shown are the distance traveled to the platform by mice injected with PBS, Tau PFFs, or IAPP-Tau PFFs. **b** Integrated time traveled in Morris water maze test. AUC, area under the curve. **c** Average swim speed of mice in three groups. **d** Probe trial results. *n* = 9 mice in PBS group, *n* = 12 mice in Tau PFFs group, *n* = 7 mice in IAPP-tau PFFs group. **e** Time spent in the novel arm in the Y-maze test. *n* = 7 mice per group. **f** Long-term potentiation (LTP) of fEPSPs was induced by 3 × TBS (4 pulses at 100 Hz, repeated three times with a 200-ms interval). The magnitude of LTP and synaptic transmission was assessed by input/output (I/O). **g** Representative fEPSPs of hippocampal slices prepared from mice in three groups. *n* = 3 mice per group. **h** Electron microscopy of synapses (top) and magnification (below). Scale bar, 1 μm in the top panel, 200 nm in the lower panel. **i** The number of synaptic clefts. **j** Postsynaptic density. **k** Length of the active zone. **l** Width of synaptic clefts. *n* = 10–15 slices per group. **m, n** Golgi staining of dendritic spines of hippocampal slides. Scale bar, 20 μm. *n* = 10 slices per group. **o** Western blot analysis of synaptic markers in the ipsilateral hippocampus of tau P301S mice. **p-r** Quantification for synaptophysin (**p**), synapsin I (**q**), and PSD95 (**r**), *n* = 3 mice per group. Data are presented as means ± SEM. One-way ANOVA followed by Tukey’s post hoc test. *P < 0.05, **P < 0.01, ***P < 0.001. n.s. not significant

Synaptic degeneration is the early event during the onset of AD, and is considered to be the basis for cognitive impairments [28]. To illustrate the effect of IAPP-tau PFFs on the memory of tau P301S mice, we performed the Morris water maze test. During the training phase, the swim distance and latency to find the platform were progressively decreased in the control mice, demonstrating a learning effect. Mice injected with tau PFFs showed learning deficits compared to the control mice. Mice injected with IAPP-tau PFFs showed the most severe learning deficits (Fig. 6a, b). The mice in different groups displayed comparable swim speeds (Fig. 6c), indicating no effect of PFFs on motor function. In the probe trial, mice injected with IAPP-tau PFFs showed the most severely impaired memory retention, as illustrated by the lower percentage of time spent in the target quadrant (Fig. 6d). Similarly, the mice injected with the IAPP-tau PFFs in the Y-maze test also showed the most severe deficits in special memory (Fig. 6e). These results indicate that IAPP-tau PFFs induce more severe AD-like cognitive impairments in vivo.

The long-term potentiation (LTP) of field excitatory postsynaptic potential (fEPSP) in the hippocampal CA1 region, which represents synaptic plasticity, was also diminished in mice injected with IAPP-tau PFFs (Fig. 6f, g), which is consistent with the results of the Morris water maze and Y-maze tests. Electron microscopy found that the density of synapses was substantially reduced in the IAPP-tau PFFs group compared with the tau PFFs group. Furthermore, the synaptic structure in the hippocampal region of the mice in the IAPP-tau PFFs group was disorganized and broken, indicating synaptic degeneration. The post-synaptic membrane had thin, discontinuous and slightly sparse dense areas (Fig. 6h-l). Furthermore, Golgi staining showed a substantial reduction in the number of dendritic spines in the IAPP-tau PFFs group (Fig. 6m, n). Consistent with the morphological results, Western blot showed that the expression of synaptic markers including synaptophysin, synapsin I, and PSD95 was reduced in mice injected with tau PFFs and IAPP-tau PFFs, indicating more severe loss of synapses. The IAPP-tau PFFs group showed more severe decrease of synaptic markers in both the ipsilateral hippocampus and cortex when compared with the tau PFFs group (Fig. 6o-r, Fig. S9e, f). These results indicate that the IAPP-tau PFFs induce more severe synaptic dysfunction and cognitive impairments in a mouse model of tauopathy.

## Discussion

In the present study, we have identified that the T2DM-associated peptide IAPP binds tau, promotes its aggregation into a more toxic strain, and enhances tau pathology in AD. IAPP interacts with the tau RD, and promotes the amyloid aggregation of tau. The resultant new IAPP-tau PFFs showed much stronger seeding activity and neurotoxicity in cultured neurons. IAPP-tau PFFs accelerated the propagation of tau pathology, induced more severe synapse loss and cognitive deficits in tau P301S transgenic mice. These findings show the role of IAPP in the onset and progression of tauopathies, and provide a mechanistic connection between T2DM and AD. IAPP shows direct interaction with tau and forms IAPP-tau mixed PFFs that were labeled with both IAPP and tau antibodies. The seeding activity of the mixed PFFs was much stronger than tau PFFs when equal amounts of PFFs were used in the in vitro and in vivo experiments. Thus, the enhanced seeding activity of IAPP-tau mixed PFFs is due to the synergy of IAPP and tau, but not simply an add-on effect.

Both AD and T2DM belong to protein deposition diseases. The incidence of AD increases in patients with T2DM [4], suggesting that there may be some pathological interaction between the two diseases. Recent reports found remarkable structural similarities between IAPP and Aβ fibrils by cryogenic electron microscopy, suggesting a possible cross-seeding between IAPP and Aβ [29–31]. Synthetic Aβ aggregates intravenously injected into human IAPP transgenic mice triggered IAPP amyloid formation, accelerated pancreatic pathology, supporting the cross-seeding interaction between these peptides [32]. However, it remains unknown whether IAPP facilitates tau aggregation. Given that both IAPP and tau are amyloidogenic and prone to aggregate into amyloid deposits, IAPP may interact with tau and enhance tau pathology. Immunofluorescence staining and co-immunoprecipitation identified the colocalization of p-tau with IAPP in the human AD brain. The levels of IAPP are higher in CSF from AD patients than that from control subjects. Furthermore, in vitro experiments found that IAPP interacts with tau and promotes the formation of the IAPP-tau mixed PFFs. We further characterized the IAPP-tau mixed fibrils by various biochemical and biological metrics, including electron microscope morphology, seeding activity in dividing cells and neurons, detergent solubility, neurotoxicity, and limited proteolysis. All these experiments indicate that IAPP triggers the formation of a pathological IAPP-tau strain, which is different from tau fibrils.

IAPP PFFs induced tau phosphorylation and neuronal injury in cultured primary neurons from tau P301S mice but not from tau knockout mice, suggesting that the effect of IAPP PFFs induced neurodegeneration is tau-dependent. The effect of IAPP-tau PFFs on tau pathology and cognitive impairments was further determined by injection of the PPFs into the hippocampus of tau P301S mice. Mice injected with IAPP-tau PFFs showed accelerated propagation of tau pathology when compared to mice injected with tau PFFs alone. Tau phosphorylation is much higher in most of the brain areas in the IAPP-tau PFFs group compared to the tau PFFs group. Thus, IAPP-tau PFFs facilitate the propagation of tau pathology in vivo. Consistent with this notion, synaptic dysfunction, neuronal loss, and neuroinflammation were also more evident in mice injected with IAPP-tau PFFs. Interestingly, tau phosphorylation was not significantly different in areas including ipsilateral dentate gyrus and retrosplenial cortex, and contralateral retrosplenial cortex 3 months post PFFs injection. This seems to be caused by the fact that tau phosphorylation was saturated in these areas in both groups.

IAPP is mainly expressed in the pancreas, but not in the brain. However, we found the presence of IAPP in brain sections from AD patients, but not that from non-AD subjects. This is consistent with the previous reports that IAPP is detected in the brain parenchyma and CSF of AD patients [32, 33]. We detected no IAPP mRNA expression in the AD brain, suggesting IAPP in the brain is from the peripheral system. The function of BBB is impaired in the elderly, which may mediate the transition of IAPP into the brain. It has been reported that circulating IAPP can pass through the BBB [34]. We found that intravenously injected IAPP entered into the mouse brain, and was taken up by neurons and microglia. This provides the possibility for IAPP to interact with tau and contribute to AD pathogenesis.

Different tauopathies such as AD, progressive supranuclear palsy, corticobasal degeneration, and Pick disease show distinct stereotyped progression of pathology, indicating the presence of different strains [35]. It has been found that tau fibrils isolated from different patients show distinct morphologies and biochemical characteristics [36]. However, the factors that initiate the formation of different strains remain elusive. Here we show that in the presence of IAPP, tau aggregates into more toxic fibrils compared to the tau fibrils. This is in agreement with the fact that diabetes and prediabetes increased the risk of conversion from amnestic cognitive impairment to dementia [37–39].

## Conclusions

In conclusion, our data indicate that IAPP contributes to the aggregation of tau, promoting its aggregation into a new strain with pronounced neurotoxicity. IAPP and tau may interact by cross-seeding, providing a potential new mechanistic explanation for the higher risk of AD in individuals affected by insulin resistance or T2DM. This is consistent with the previous experiments addressing the potential role of diabetes in dementia [38] and in exacerbating tau pathology [40, 41]. Antidiabetic drugs have been reported to reduce tau deposits and improve cognitive function both in mouse models and human subjects [42, 43]. Likewise, compounds inhibiting the aggregation and/or stimulating the elimination of IAPP aggregates in T2DM may have the potential to treat AD [44]. Indeed, suppressing the secretion of amylin protected a rodent model against AD-associated phenotypes [33]. Moreover, treatment with the IAPP receptor antagonist has been shown to improve cognitive functions and reduce AD pathology in mouse models of AD [45]. However, intraperitoneal injection of IAPP or its analog pramlintide was reported to remove Aβ from the brain and reduce cognitive impairment in AD animal models [46, 47], indicating a potential protective role of IAPP against Aβ pathology. Thus, further studies are needed to investigate the molecular mechanisms mediating the beneficial and detrimental effects of IAPP, and develop specific therapeutic targets to prevent the development of AD.

Here we show that the formation of tau aggregate can be accelerated by heterogeneous seeds of IAPP. This cross-seeding study verified the fundamental disease mechanism whereby strain-specific differences govern seeding, propagation, and specific regional vulnerability in tauopathies. Further studies are needed to explore whether blocking the interaction of tau and IAPP alleviates AD pathology, and to illustrate whether IAPP-tau fibrils can serve as a biomarker for the early diagnosis of AD, especially those with T2DM.

## Supplementary Information

**Additional file 1: Figure S1.** IAPP interacts with p-tau. **(a)** Representative co-immunostaining of IAPP and neuronal/glial markers in AD brain slices. Scale bar, 20 μm. **(b)** Co-immunostainings of IAPP and p-tau (AT8) in brain sections of AD patients. Scale bar, 20 μm. **(c)** GST pull-down analysis indicating the interaction between IAPP and tau in vitro. **(d)** GST pull-down analysis showing that IAPP interacts with tau RD in vitro. Experiments were independently performed three times. **(e, f)** Uptake of intravenously injected FITC-IAPP PFFs by neurons (e) and microglia (f) in wild-type mice. (**g, h**) Quantification of intravenously injected FITC-IAPP PFFs by neurons (g) and microglia (h) in wild-type and tau P301S mice. Scale bars, 20 μm. *n* = 10 slices from 5 mice. Bars represent means ± SEM. Unpaired Student’s *t-*test. ***P < 0.001.

**Additional file 2: Figure S2.** IAPP accelerates tau fibrillization in vitro. **(a)** ThT fluorescence assay of tau fibrillization in the presence of IAPP PFFs (*n* = 3 independent samples). **(b)** Immuno-EM analysis of the IAPP-tau fibrils. The fibrils were stained using antibodies coupled to gold nanoparticles. IAPP was detected with an antibody coupled to 8 nm gold particles (arrow), while His-K18 was labeled with 4 nm gold particles (arrowheads). Scale bar, 50 nm. **(c, d)** Western blot showing the amount of tau PFFs and IAPP-tau PFFs. **(e, f)** Western blot showing the uptake of tau PFFs and IAPP-tau PFFs by cells after co-incubation for 24 h. *n* = 3 independent samples in each group. Data are presented as means ± SEM. One-way ANOVA followed by Tukey’s post hoc test. n.s. not significant.

**Additional file 3: Figure S3.** IAPP PFFs induce tau phosphorylation and neurotoxicity in a tau-dependent manner in vitro. **(a-d)** Representative immunostaining and quantification of AT8 (a-b) and TUNEL assay (c-d) in primary neurons from tau P301S mice treated with PBS or IAPP PFFs for 5 days. *n* = 10 slices from 3 independent experiments. Scale bar, 20 μm. Bars represent means ± SEM. Unpaired Student’s *t-*test. ***P < 0.001. **(e, f)** Representative immunostaining and quantification of TUNEL staining in cultured tau-knockout neurons treated with PBS or IAPP PFFs for 5 days. Experiments were independently performed three times. Fifty visual fields (Fig. S3b) and 150 cells (Fig. S3d, S3f) from 10 slices were counted in each group. Scale bar, 20 μm. Bars represent means ± SEM. One-way ANOVA followed by Tukey’s post hoc test. n.s. not significant. ***P < 0.001.

**Additional file 4: Figure S4.** IAPP-tau PFFs induce tau phosphorylation in vitro. **(a-d)** Representative immunostaining and quantification of p-tau Ser202 (a, b) and p-tau Ser396 (c, d) in primary neurons treated with PBS, Tau PFFs, or IAPP-Tau PFFs for 5 days. *n* = 10–11 slices from 3 independent experiments. **(e, f)** Western blot analysis and quantification of AT8 in primary cortical neurons transduced with tau PFFs and IAPP-tau PFFs. *n* = 4 independent samples. Bars represent means ± SEM. One-way ANOVA followed by Tukey’s post hoc test. Scale bar, 20 μm. *P < 0.05, **P < 0.01, ***P < 0.001.

**Additional file 5: Figure S5.** IAPP PFFs induce tau pathology and cognitive decline in vivo*.* (**a, b**) Representative images of AT8 staining in tissue sections from the hippocampus, dentate gyrus, and cortex of tau P301S mice one month (a) and three months (b) after the injection of PBS or IAPP PFFs. Scale bar, 200 μm in (a) and (b) upper panel, 100 μm in (a) and (b) middle panel, 50 μm in (a) and (b) lower panels. **(c-e)** Quantification of AT8 pathology of ipsilateral and contralateral sides in the hippocampus (c), dentate gyrus (d), and entorhinal cortex (e). *n* = 4 mice per group. Data are presented as means ± SEM. Unpaired Student’s *t*-test, *P < 0.05, **P < 0.01, ***P < 0.001, n.s. not significant. **(f)** Spatial memory was assessed by the Morris water maze test. Shown are distance traveled to the platform by mice injected with PBS or IAPP PFFs. **(g)** Integrated time traveled in Morris water maze test. AUC, area under the curve. **(h)** Swim speed of mice in three groups. **(i)** Probe trial results. *n* = 8 mice in PBS and IAPP PFFs group, respectively. **(j)** Time spent in the novel arm in the Y-maze test. *n* = 8 mice per group. **(k)** Western blot analysis of synaptic markers in the hippocampus of tau P301S mice. **(l)** Quantification of synaptophysin, synapsin I, and PSD95. *n* = 3 mice per group. Data are presented as means ± SEM. Unpaired Student’s *t*-test, *P < 0.05, **P < 0.01, ***P < 0.001. n.s. not significant.

**Additional file 6: Figure S6.** IAPP-Tau PFFs promote tau transmission in vivo*.* **(a, b)** Representative images of AT8 p-tau pathology in tissue sections (posterior hippocampal level) from the hippocampus, CA1, CA3, entorhinal cortex, and auditory cortex of tau P301S mice one (a) or three (b) months after the injection of either PBS, Tau PFFs or IAPP-Tau PFFs. **(c-l)** Quantification of AT8 p-tau pathology of the ipsilateral and contralateral sides of the hippocampus (c, d), CA1 (e, f), CA3 (g, h), entorhinal cortex (i, j), and auditory cortex (k, l). Data are presented as means ± SEM. One-way ANOVA followed by Tukey’s post hoc test (*n* = 4 mice per group). Scale bar, 200 μm for (a) and (b) upper panel, 50 μm for (a) and (b) lower panels. *P < 0.05, **P < 0.01, ***P < 0.001.

**Additional file 7: Figure S7.** IAPP-Tau PFFs promote the propagation of tau pathology in vivo. **(a, b)** Representative images of p-tau (AT8) in the hippocampus of tau P301S mice 1 month (a) and 3 months (b) after the injection of PBS, IAPP PFFs, tau PFFs and IAPP-tau PFFs. Scale bar, 200 μm. **(c, d)** Quantification of p-tau in the ipsilateral and contralateral hippocampus 1 month (c) and 3 months (d) after injection. *n* = 4 mice per group. Data are presented as means ± SEM. One-way ANOVA followed by Tukey’s post hoc test. *P < 0.05, **P < 0.01, ***P < 0.001. n.s. not significant.

**Additional file 8: Figure S8.** IAPP-Tau PFFs promote tau phosphorylation in vivo*.* **(a)** Immunoblot analysis of p-tau in the cortex of tau P301S mice injected with either PBS, Tau PFFs, or IAPP-Tau PFFs. **(b)** Statistical analysis of AT8, AT100, and tau5 expression in (a). Data are presented as means ± SEM. One-way ANOVA followed by Tukey’s post hoc test (*n* = 4 mice per group). *P < 0.05, **P < 0.01, ***P < 0.001, n.s. not significant. **(c, d)** Cortex samples were sequentially extracted with 1% Triton X-100 (TX-soluble) and 2% SDS (TX-insoluble) from mice injected with either PBS, Tau PFFs, or IAPP-Tau PFFs. Representative Western blot (c) and quantification (d) are presented. Data are presented as means ± SEM. One-way ANOVA followed by Tukey’s post hoc test (*n* = 3 mice per group). *P < 0.05, **P < 0.01, ***P < 0.001, n.s. not significant.

**Additional file 9: Figure S9.** IAPP-Tau PFFs induce synaptic dysfunction and neuroinflammation in vivo*.* **(a-d)** Immunostaining and quantification of microglia marker IBA1 (a, b) and astrocyte marker GFAP (c, d) 3 months after the mice were injected with Tau PFFs or IAPP-Tau PFFs (*n* = 4 mice per group). Scale bar, 50 μm for lower panel, 20 μm for magnification. Data are presented as means ± SEM. One-way ANOVA followed by Tukey’s post hoc test was used. **(e)** Western blot analysis of synaptic markers in the cortex of tau P301S mice. **(f)** Quantification of synaptophysin, synapsin I, and PSD95 in (e). Data are presented as means ± SEM. One-way ANOVA followed by Tukey’s post hoc test (*n* = 3 mice per group).*P < 0.05, **P < 0.01, ***P < 0.001, n.s. not significant.

**Additional file 10: Table S1.** Clinical information of human post-mortem tissues in Fig. 1a, c, e-g**,** and Fig. S1a-b.

**Additional file 11: Table S2.** Clinical information of AD and control CSF in Fig. 1d.

## Data Availability

The datasets used and analyzed during the current study are available from the corresponding author on reasonable request.

## Abbreviations

AD: Alzheimer’s disease
T2DM: Type 2 diabetes
IAPP: Islet amyloid polypeptide
BBB: Blood-brain barrier
MCI: Mild cognitive impairment
CSF: Cerebrospinal fluid
Aβ: Amyloid-β
PFFs: Pre-formed fibrils
TEM: Transmission electron microscopy
ThT: Thioflavin T
ELISA: Enzyme-linked immunosorbent assay
PK: Proteinase K
MPI: Months-post-injection
LTP: Long-term potentiation
fEPSP: Field excitatory postsynaptic potential
