# Supplementary figures and images for: Islet amyloid polypeptide cross-seeds tau and drives the neurofibrillary pathology in Alzheimer’s disease
Source: Mol Neurodegener. 2022 Jan 29;17:12. doi: 10.1186/s13024-022-00518-y (PMC8800231; doi:10.1186/s13024-022-00518-y)

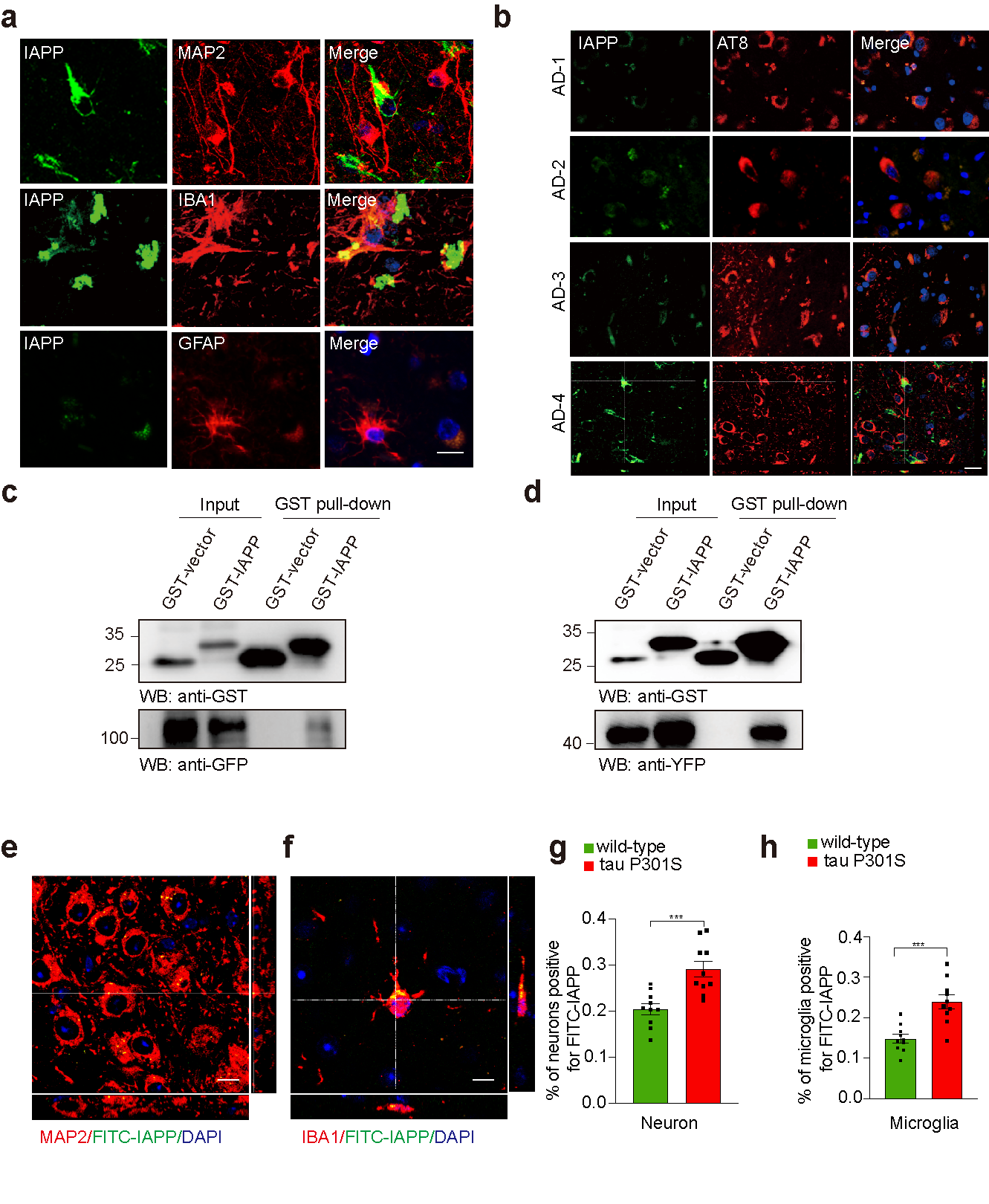

Supplement: Supplementary file 1 — Additional file 1: Figure S1. IAPP interacts with p-tau. (a) Representative co-immunostaining of IAPP and neuronal/glial markers in AD brain slices. Scale bar, 20 μm. (b) Co-immunostainings of IAPP and p-tau (AT8) in brain sections of AD patients. Scale bar, 20 μm. (c) GST pull-down analysis indicating the interaction between IAPP and tau in vitro. (d) GST pull-down analysis showing that IAPP interacts with tau RD in vitro. Experiments were independently performed three times. (e, f) Uptake of intravenously injected FITC-IAPP PFFs by neurons (e) and microglia (f) in wild-type mice. (g, h) Quantification of intravenously injected FITC-IAPP PFFs by neurons (g) and microglia (h) in wild-type and tau P301S mice. Scale bars, 20 μm. n = 10 slices from 5 mice. Bars represent means ± SEM. Unpaired Student’s t-test. ***P < 0.001. [file 13024_2022_518_MOESM1_ESM.tif]

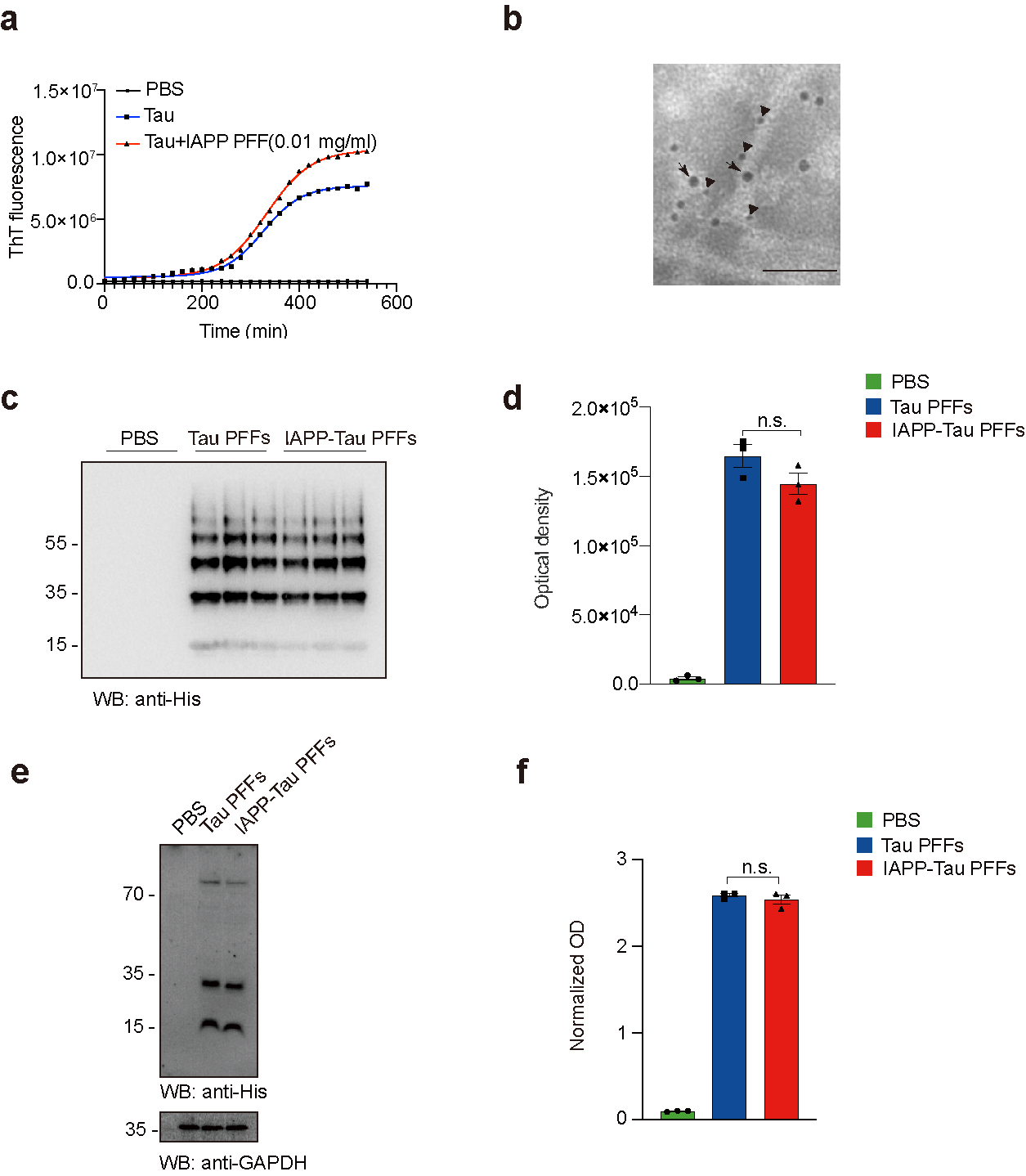

Supplement: Supplementary file 2 — Additional file 2: Figure S2. IAPP accelerates tau fibrillization in vitro. (a) ThT fluorescence assay of tau fibrillization in the presence of IAPP PFFs (n = 3 independent samples). (b) Immuno-EM analysis of the IAPP-tau fibrils. The fibrils were stained using antibodies coupled to gold nanoparticles. IAPP was detected with an antibody coupled to 8 nm gold particles (arrow), while His-K18 was labeled with 4 nm gold particles (arrowheads). Scale bar, 50 nm. (c, d) Western blot showing the amount of tau PFFs and IAPP-tau PFFs. (e, f) Western blot showing the uptake of tau PFFs and IAPP-tau PFFs by cells after co-incubation for 24 h. n = 3 independent samples in each group. Data are presented as means ± SEM. One-way ANOVA followed by Tukey’s post hoc test. n.s. not significant. [file 13024_2022_518_MOESM2_ESM.tif]

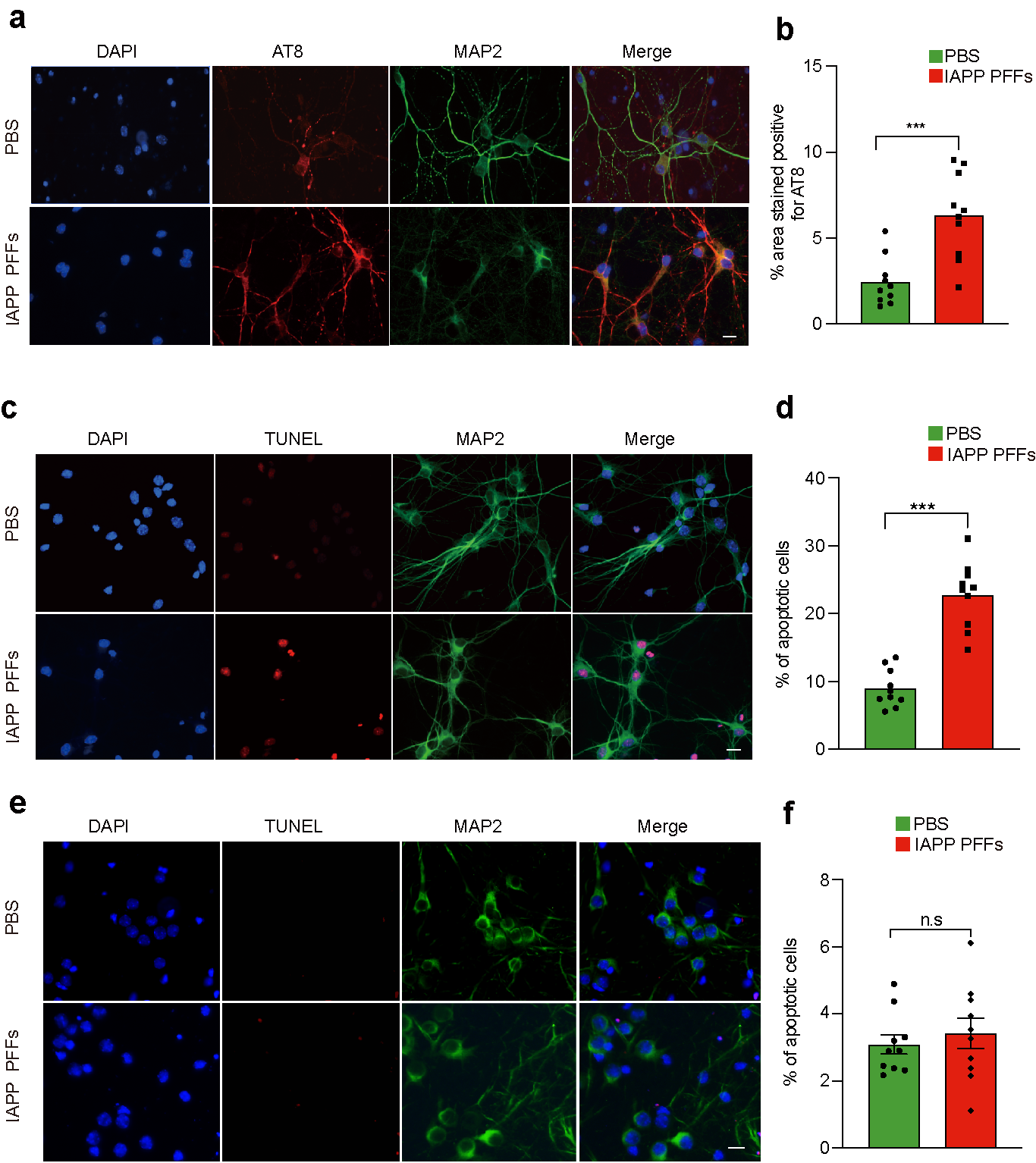

Supplement: Supplementary file 3 — Additional file 3: Figure S3. IAPP PFFs induce tau phosphorylation and neurotoxicity in a tau-dependent manner in vitro. (a-d) Representative immunostaining and quantification of AT8 (a-b) and TUNEL assay (c-d) in primary neurons from tau P301S mice treated with PBS or IAPP PFFs for 5 days. n = 10 slices from 3 independent experiments. Scale bar, 20 μm. Bars represent means ± SEM. Unpaired Student’s t-test. ***P < 0.001. (e, f) Representative immunostaining and quantification of TUNEL staining in cultured tau-knockout neurons treated with PBS or IAPP PFFs for 5 days. Experiments were independently performed three times. Fifty visual fields (Fig. S3b) and 150 cells (Fig. S3d, S3f) from 10 slices were counted in each group. Scale bar, 20 μm. Bars represent means ± SEM. One-way ANOVA followed by Tukey’s post hoc test. n.s. not significant. ***P < 0.001. [file 13024_2022_518_MOESM3_ESM.tif]

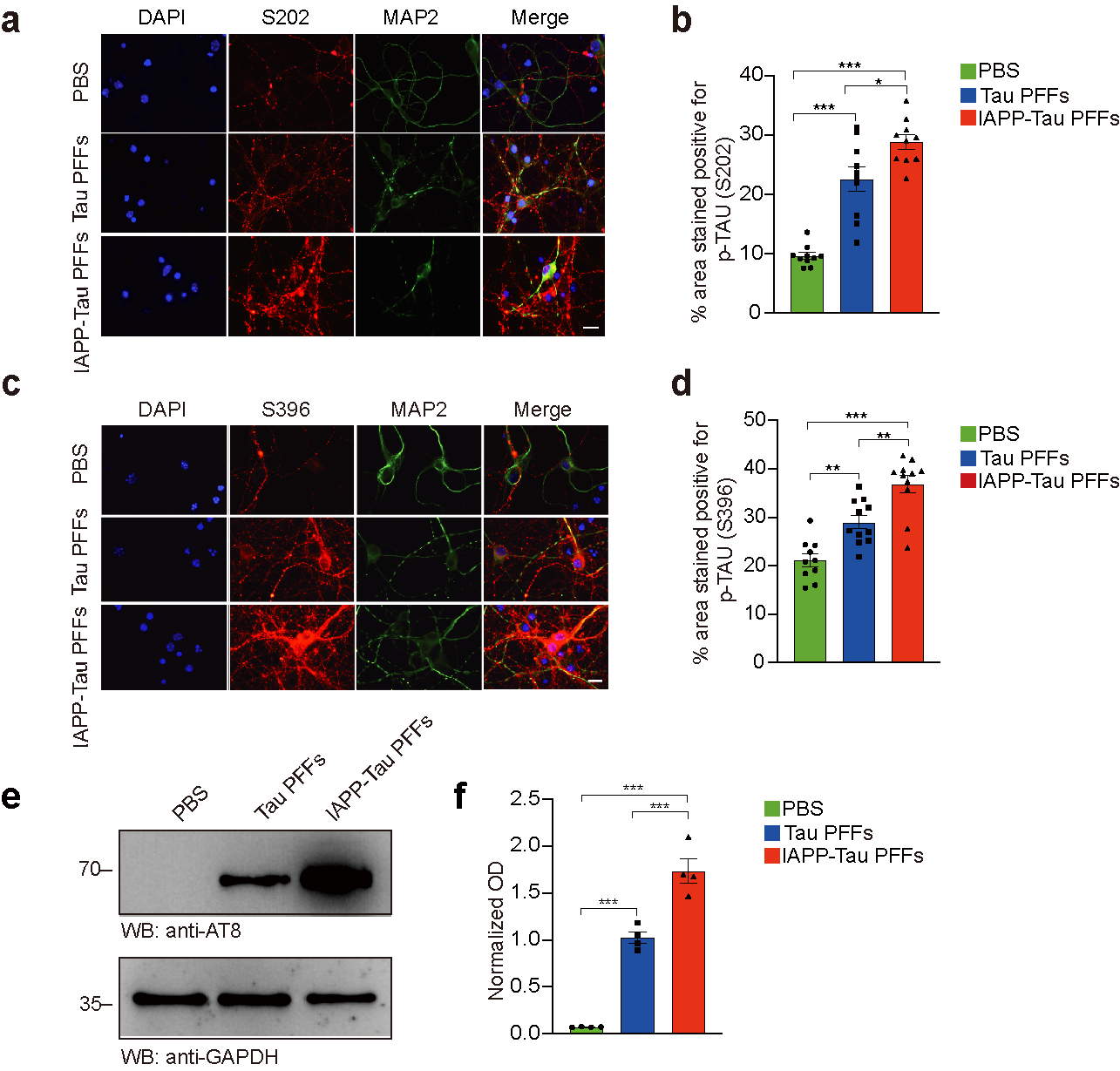

Supplement: Supplementary file 4 — Additional file 4: Figure S4. IAPP-tau PFFs induce tau phosphorylation in vitro. (a-d) Representative immunostaining and quantification of p-tau Ser202 (a, b) and p-tau Ser396 (c, d) in primary neurons treated with PBS, Tau PFFs, or IAPP-Tau PFFs for 5 days. n = 10–11 slices from 3 independent experiments. (e, f) Western blot analysis and quantification of AT8 in primary cortical neurons transduced with tau PFFs and IAPP-tau PFFs. n = 4 independent samples. Bars represent means ± SEM. One-way ANOVA followed by Tukey’s post hoc test. Scale bar, 20 μm. *P < 0.05, **P < 0.01, ***P < 0.001. [file 13024_2022_518_MOESM4_ESM.tif]

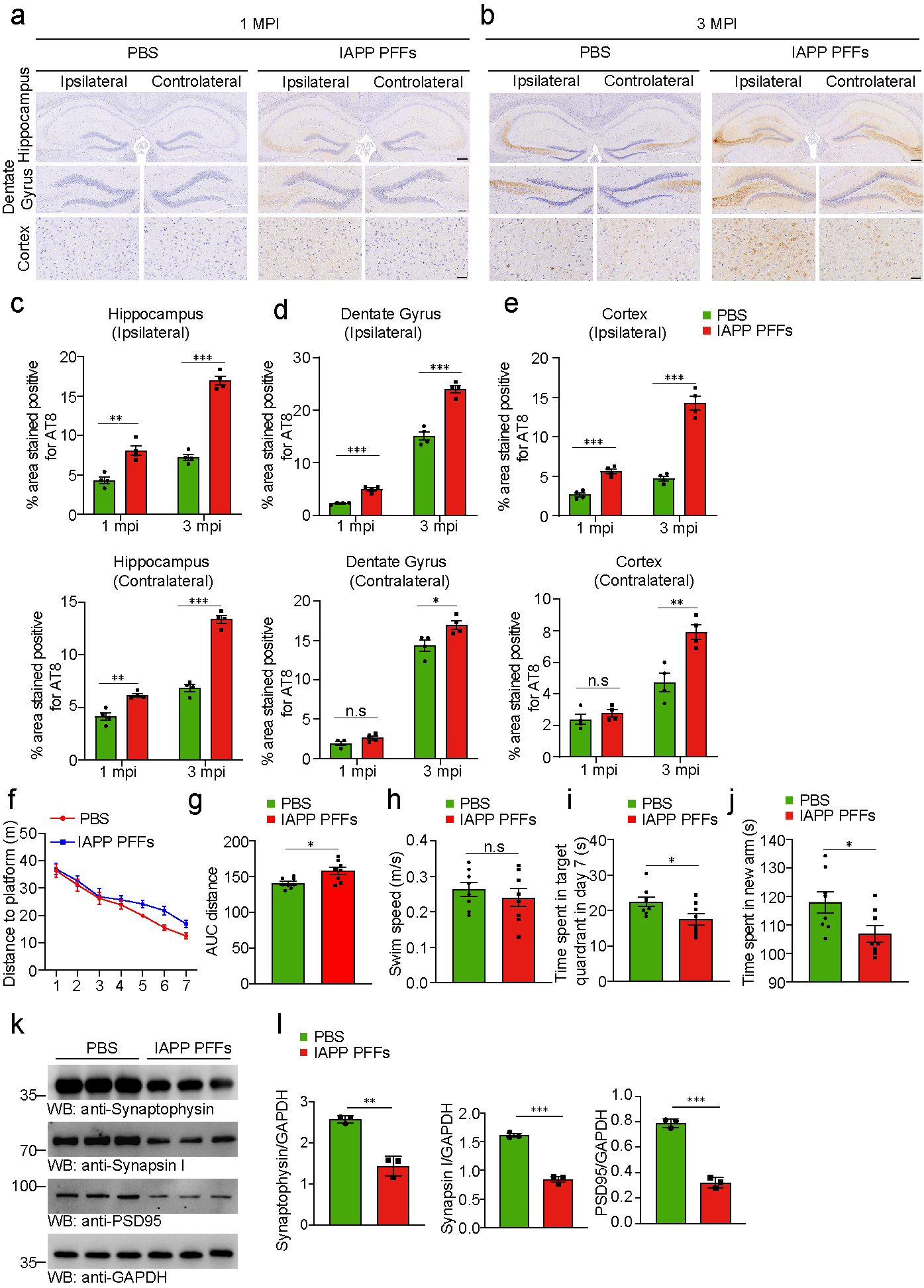

Supplement: Supplementary file 5 — Additional file 5: Figure S5. IAPP PFFs induce tau pathology and cognitive decline in vivo. (a, b) Representative images of AT8 staining in tissue sections from the hippocampus, dentate gyrus, and cortex of tau P301S mice one month (a) and three months (b) after the injection of PBS or IAPP PFFs. Scale bar, 200 μm in (a) and (b) upper panel, 100 μm in (a) and (b) middle panel, 50 μm in (a) and (b) lower panels. (c-e) Quantification of AT8 pathology of ipsilateral and contralateral sides in the hippocampus (c), dentate gyrus (d), and entorhinal cortex (e). n = 4 mice per group. Data are presented as means ± SEM. Unpaired Student’s t-test, *P < 0.05, **P < 0.01, ***P < 0.001, n.s. not significant. (f) Spatial memory was assessed by the Morris water maze test. Shown are distance traveled to the platform by mice injected with PBS or IAPP PFFs. (g) Integrated time traveled in Morris water maze test. AUC, area under the curve. (h) Swim speed of mice in three groups. (i) Probe trial results. n = 8 mice in PBS and IAPP PFFs group, respectively. (j) Time spent in the novel arm in the Y-maze test. n = 8 mice per group. (k) Western blot analysis of synaptic markers in the hippocampus of tau P301S mice. (l) Quantification of synaptophysin, synapsin I, and PSD95. n = 3 mice per group. Data are presented as means ± SEM. Unpaired Student’s t-test, *P < 0.05, **P < 0.01, ***P < 0.001. n.s. not significant. [file 13024_2022_518_MOESM5_ESM.tif]

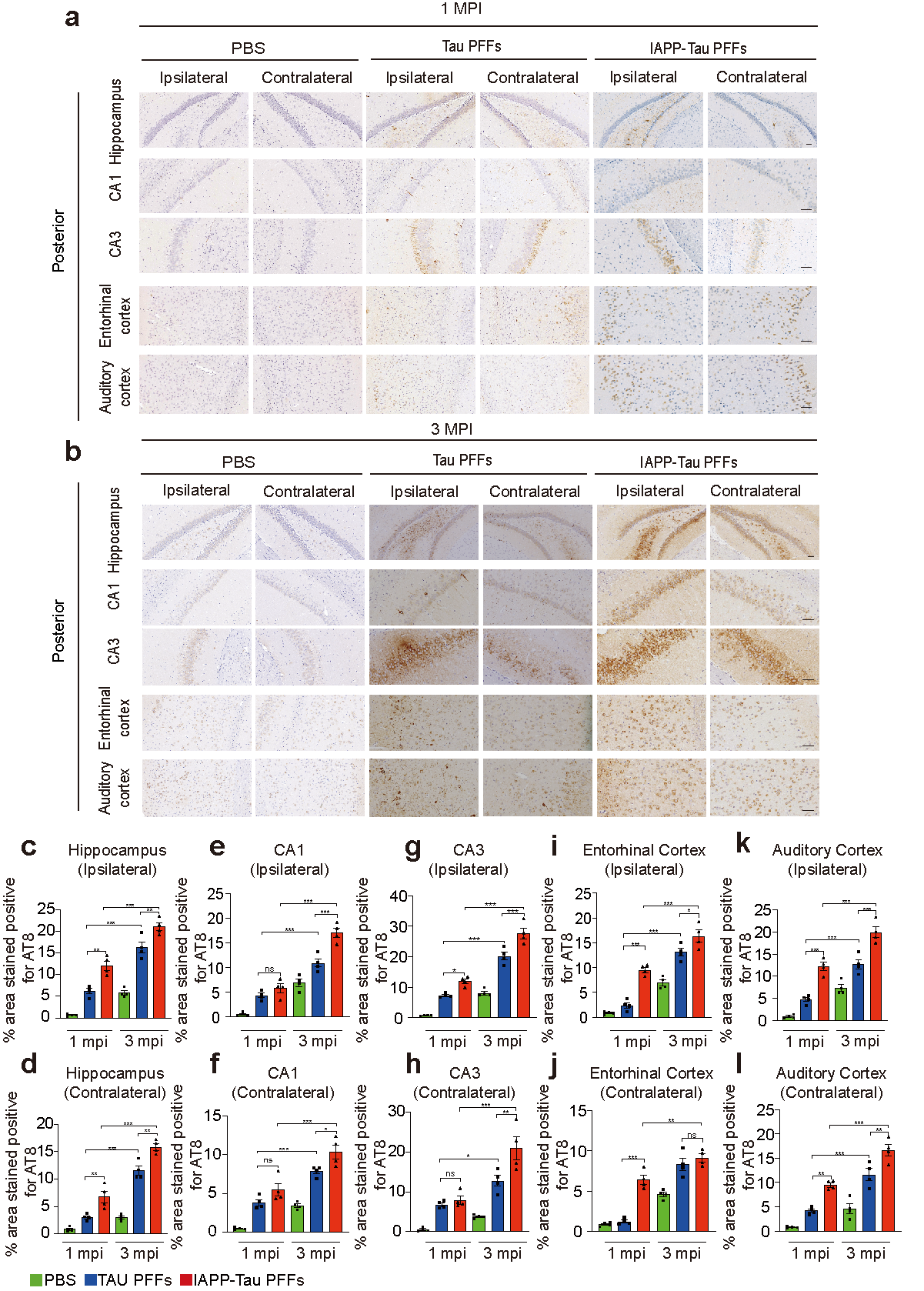

Supplement: Supplementary file 6 — Additional file 6: Figure S6. IAPP-Tau PFFs promote tau transmission in vivo. (a, b) Representative images of AT8 p-tau pathology in tissue sections (posterior hippocampal level) from the hippocampus, CA1, CA3, entorhinal cortex, and auditory cortex of tau P301S mice one (a) or three (b) months after the injection of either PBS, Tau PFFs or IAPP-Tau PFFs. (c-l) Quantification of AT8 p-tau pathology of the ipsilateral and contralateral sides of the hippocampus (c, d), CA1 (e, f), CA3 (g, h), entorhinal cortex (i, j), and auditory cortex (k, l). Data are presented as means ± SEM. One-way ANOVA followed by Tukey’s post hoc test (n = 4 mice per group). Scale bar, 200 μm for (a) and (b) upper panel, 50 μm for (a) and (b) lower panels. *P < 0.05, **P < 0.01, ***P < 0.001. [file 13024_2022_518_MOESM6_ESM.tif]

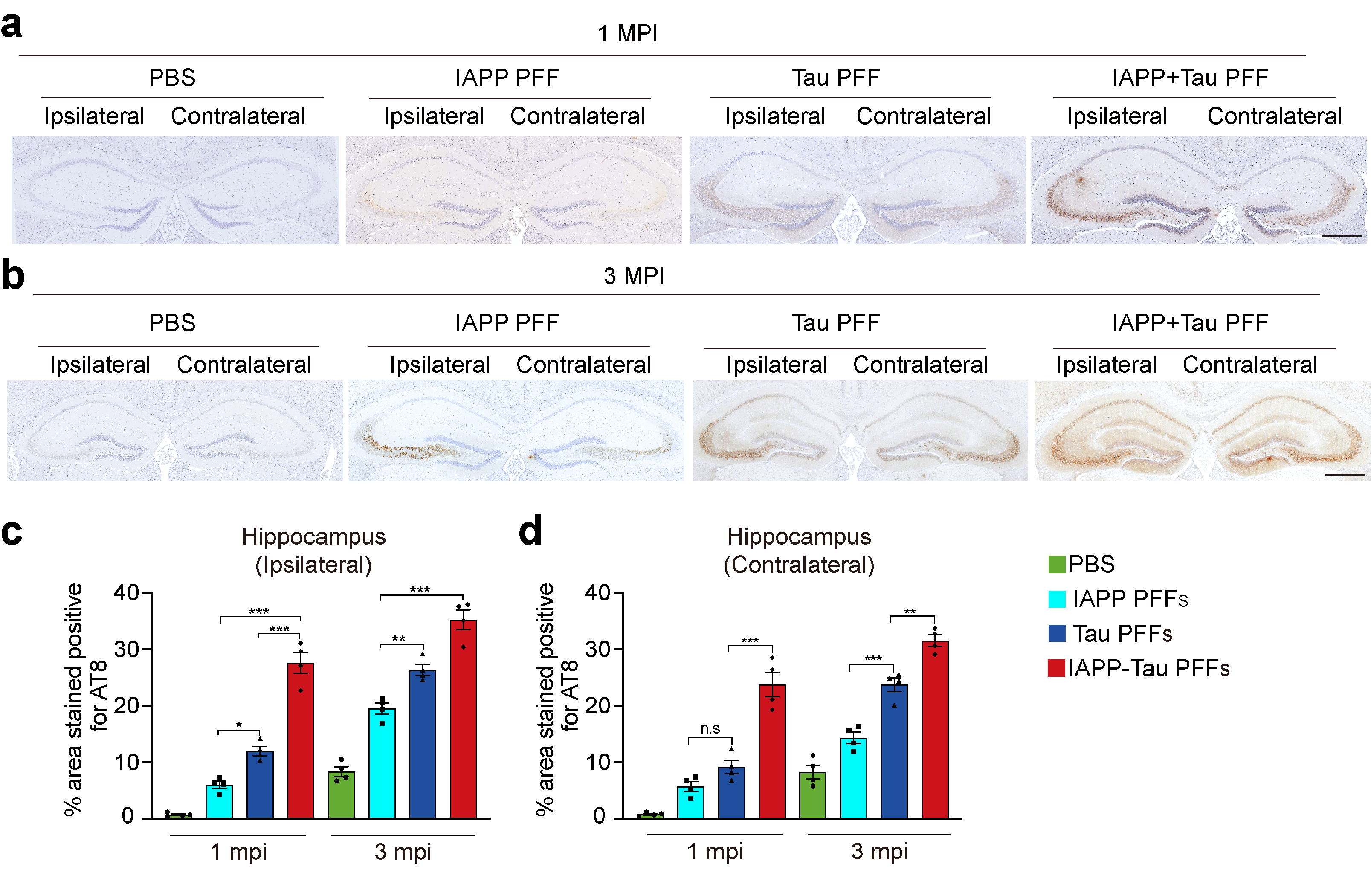

Supplement: Supplementary file 7 — Additional file 7: Figure S7. IAPP-Tau PFFs promote the propagation of tau pathology in vivo. (a, b) Representative images of p-tau (AT8) in the hippocampus of tau P301S mice 1 month (a) and 3 months (b) after the injection of PBS, IAPP PFFs, tau PFFs and IAPP-tau PFFs. Scale bar, 200 μm. (c, d) Quantification of p-tau in the ipsilateral and contralateral hippocampus 1 month (c) and 3 months (d) after injection. n = 4 mice per group. Data are presented as means ± SEM. One-way ANOVA followed by Tukey’s post hoc test. *P < 0.05, **P < 0.01, ***P < 0.001. n.s. not significant. [file 13024_2022_518_MOESM7_ESM.tif]

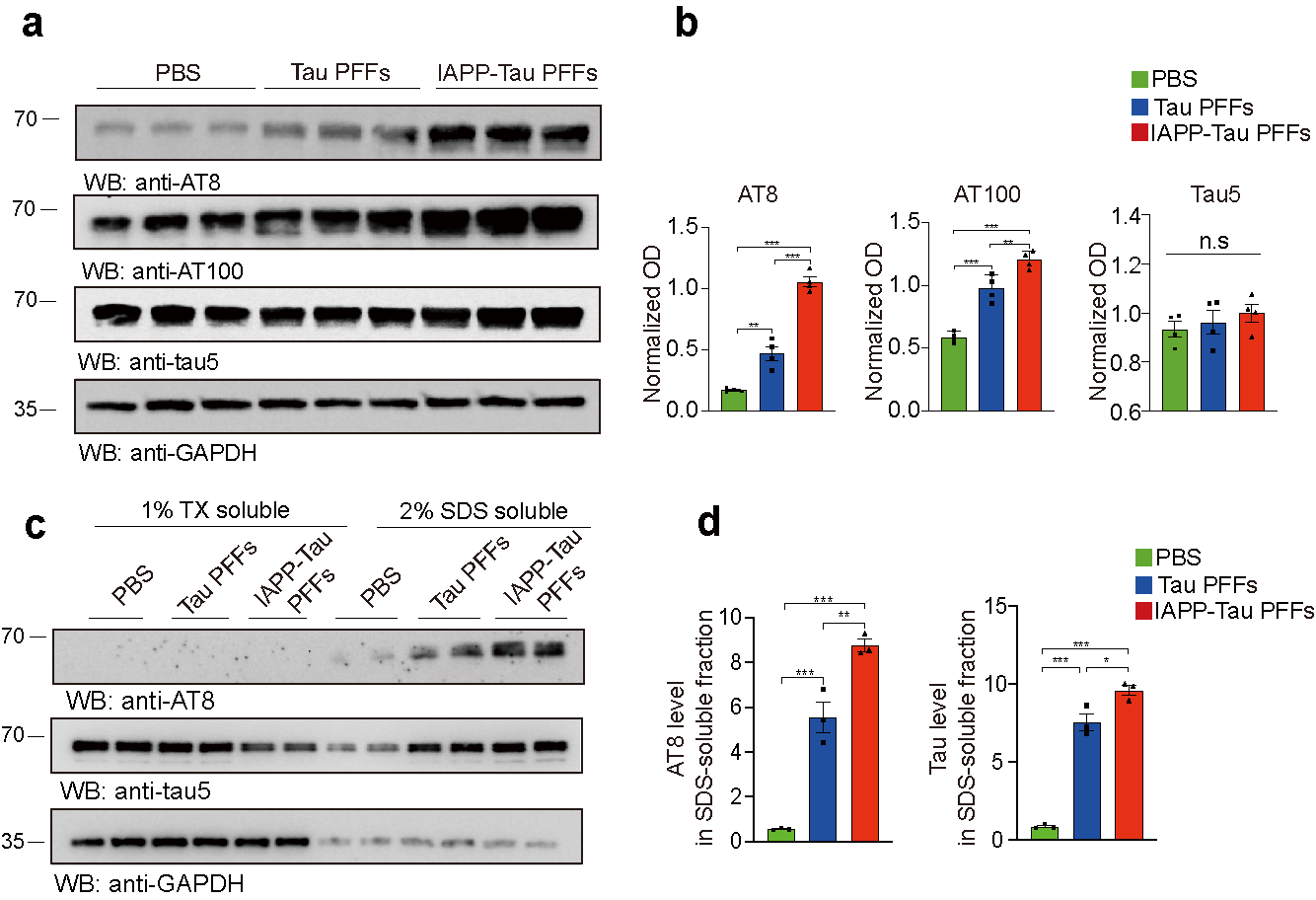

Supplement: Supplementary file 8 — Additional file 8: Figure S8. IAPP-Tau PFFs promote tau phosphorylation in vivo. (a) Immunoblot analysis of p-tau in the cortex of tau P301S mice injected with either PBS, Tau PFFs, or IAPP-Tau PFFs. (b) Statistical analysis of AT8, AT100, and tau5 expression in (a). Data are presented as means ± SEM. One-way ANOVA followed by Tukey’s post hoc test (n = 4 mice per group). *P < 0.05, **P < 0.01, ***P < 0.001, n.s. not significant. (c, d) Cortex samples were sequentially extracted with 1% Triton X-100 (TX-soluble) and 2% SDS (TX-insoluble) from mice injected with either PBS, Tau PFFs, or IAPP-Tau PFFs. Representative Western blot (c) and quantification (d) are presented. Data are presented as means ± SEM. One-way ANOVA followed by Tukey’s post hoc test (n = 3 mice per group). *P < 0.05, **P < 0.01, ***P < 0.001, n.s. not significant. [file 13024_2022_518_MOESM8_ESM.tif]

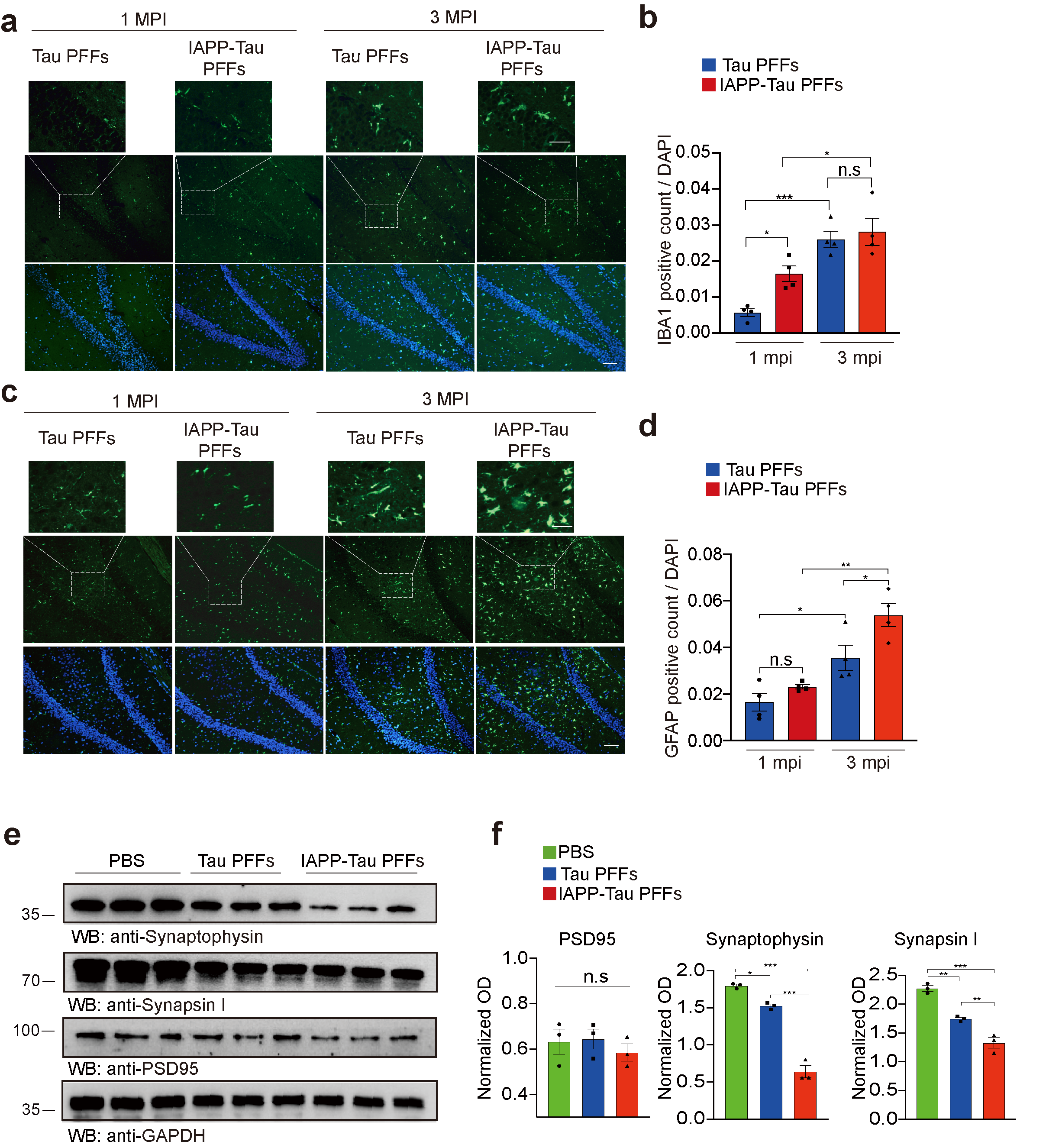

Supplement: Supplementary file 9 — Additional file 9: Figure S9. IAPP-Tau PFFs induce synaptic dysfunction and neuroinflammation in vivo. (a-d) Immunostaining and quantification of microglia marker IBA1 (a, b) and astrocyte marker GFAP (c, d) 3 months after the mice were injected with Tau PFFs or IAPP-Tau PFFs (n = 4 mice per group). Scale bar, 50 μm for lower panel, 20 μm for magnification. Data are presented as means ± SEM. One-way ANOVA followed by Tukey’s post hoc test was used. (e) Western blot analysis of synaptic markers in the cortex of tau P301S mice. (f) Quantification of synaptophysin, synapsin I, and PSD95 in (e). Data are presented as means ± SEM. One-way ANOVA followed by Tukey’s post hoc test (n = 3 mice per group).*P < 0.05, **P < 0.01, ***P < 0.001, n.s. not significant. [file 13024_2022_518_MOESM9_ESM.tif]
