# Supplementary material for: Islet amyloid polypeptide cross-seeds tau and drives the neurofibrillary pathology in Alzheimer’s disease
Source: Mol Neurodegener. 2022 Jan 29;17:12. doi: 10.1186/s13024-022-00518-y (PMC8800231; doi:10.1186/s13024-022-00518-y)
Supplement: Supplementary file 10 — Additional file 10: Table S1. Clinical information of human post-mortem tissues in Fig. 1a, c, e-g, and Fig. S1a-b. [file 13024_2022_518_MOESM10_ESM.docx]

**Table S1. Clinical information of human post-mortem tissues in Fig. 1a, 1c, 1e-g, and Fig S1a-b.**

| Group | Age at death | Sex | PMD (hr) | Braak stage |
| --- | --- | --- | --- | --- |
| AD | 70 | F | 7 | IV |
|  | 65 | M | 9 | III |
|  | 78 | F | 17 | V |
|  | 91 | F | 21 | VI |
|  | 84 | F | 34 | VI |
|  | 78 | M | 19 | V |
|  | 85 | F | 14 | VI |
|  | 82 | M | 8 | V |
|  | 73 | M | 10 | IV |
|  | 79 | F | 9 | V |
| Control | 75 | M | 16 | - |
|  | 77 | F | 11 | - |
|  | 76 | M | 13 | - |
|  | 78 | F | 7 | - |
|  | 82 | F | 10 | - |
|  | 67 | M | 6 | - |
|  | 89 | F | 12 | - |
|  | 92 | F | 18 | - |
|  | 78 | M | 13 | - |
|  | 75 | M | 21 | - |

PMD, post-mortem delay.
