# Supplementary material for: Islet amyloid polypeptide cross-seeds tau and drives the neurofibrillary pathology in Alzheimer’s disease
Source: Mol Neurodegener. 2022 Jan 29;17:12. doi: 10.1186/s13024-022-00518-y (PMC8800231; doi:10.1186/s13024-022-00518-y)
Supplement: Supplementary file 11 — Additional file 11: Table S2. Clinical information of AD and control CSF in Fig. 1d. [file 13024_2022_518_MOESM11_ESM.docx]

**Table S2. Clinical information of AD and control CSF in Fig. 1d.**

| Group | Sex | Age (year) | | Disease duration (year) | | |
| --- | --- | --- | --- | --- | --- | --- |
| AD patients (n=11) | M | 76 | | 3 | | |
|  | M | 78 | | 5 | | |
|  | M | 72 | | 5 | | |
|  | F | 75 | | 4 | | |
|  | F | 68 | | 6 | | |
|  | F | 72 | | 3 | | |
|  | M | 66 | | 2 | | |
|  | M | 65 | | 2 | | |
|  | F | 70 | | 3 | | |
|  | F | 67 | | 2 | | |
|  | F | 67 | | 3 | | |
| Control (n=22) | M | 65 | | 0 | |  |
|  | M | 68 | | 0 | |  |
|  | F | 62 | | 0 | |  |
|  | M | 70 | | 0 | |  |
|  | F | 65 | | 0 | |  |
|  | F | 71 | | 0 | |  |
|  | F | 67 | | 0 | |  |
|  | M | 67 | | 0 | |  |
|  | F | 65 | | 0 | |  |
|  | M | 69 | | 0 | |  |
|  | M | 74 | | 0 | |  |
|  | F | 72 | | 0 | |  |
|  | F | 62 | | 0 | |  |
|  | M | 64 | | 0 | |  |
|  | F | 67 | | 0 | |  |
|  | F | 76 | | 0 | |  |
|  | M | 72 | | 0 | |  |
|  | F | 65 | | 0 | |  |
|  | F | 67 | | 0 | |  |
|  | M | 66 | | 0 | |  |
|  | F | 70 | | 0 | |  |
|  | F | 71 | | 0 | |  |
| **Summarized information** | | | | | | |
| Group | Mean age ± SEM | | Female/male ratio (%) | | Mean disease  duration ± SEM |  |
| AD | 70.55 ± 1.324 | | 54.5/45.5 | | 3.455 ± 0.4126 |  |
| Control | 67.95 ± 0.7936 | | 59.1/40.9 | | 0 |  |

* Disease duration is the time since AD symptom onset.
